# Streamlined sensory motor communication through cortical reciprocal connectivity in a visually guided eye movement task

**DOI:** 10.1038/s41467-017-02501-4

**Published:** 2018-01-23

**Authors:** Takahide Itokazu, Masashi Hasegawa, Rui Kimura, Hironobu Osaki, Urban-Raphael Albrecht, Kazuhiro Sohya, Shubhodeep Chakrabarti, Hideaki Itoh, Tetsufumi Ito, Tatsuo K. Sato, Takashi R. Sato

**Affiliations:** 1 Werner Reichardt Center for Integrative Neuroscience, University of Tübingen, 72076 Tübingen, Germany; 2 Department of Mental Disorder Research, National Institute of Neuroscience, National Center of Neurology and Psychiatry, Tokyo, 187-8502 Japan; 3 Graduate School of Science and Engineering, Saga University, Saga, 840-8502 Japan; 4 Department of Anatomy, Kanazawa Medical University, Ishikawa, 920-0293 Japan; 5 Institute of Neuroscience, Technical University of Munich, 80802 Munich, Germany; 6 PRESTO, Japan Science and Technology Agency, Saitama, 332-0012 Japan; 7 Present Address: Department of Physiology, Tokyo Women’s Medical University, Tokyo, 162-8666 Japan

## Abstract

Cortical computation is distributed across multiple areas of the cortex by networks of reciprocal connectivity. However, how such connectivity contributes to the communication between the connected areas is not clear. In this study, we examine the communication between sensory and motor cortices. We develop an eye movement task in mice and combine it with optogenetic suppression and two-photon calcium imaging techniques. We identify a small region in the secondary motor cortex (MO_s_) that controls eye movements and reciprocally connects with a rostrolateral part of the higher visual areas (V_RL/A/AL_). These two regions encode both motor signals and visual information; however, the information flow between the regions depends on the direction of the connectivity: motor information is conveyed preferentially from the MO_s_ to the V_RL/A/AL_, and sensory information is transferred primarily in the opposite direction. We propose that reciprocal connectivity streamlines information flow, enhancing the computational capacity of a distributed network.

## Abstract (web summary)

Reciprocal connectivity enables tightly coupled information processing across cortical areas. Here the authors develop a visual oculomotor task in mice, identify a small motor area required for it, and demonstrate selective exchange of sensory and motor information between the motor and sensory areas.

## Introduction

The cerebral cortex contains many anatomically defined areas^1^, each of which is proposed to have its preferred functional specialization^2^. These areas integrate with each other through long-range connections to perform complex yet efficient cortical computations^3^. For sensory processing, these connections link separate cortical areas and allow them to operate as a distributed hierarchical network^2,4–6^. Although the wiring patterns behind these connections have been subject to intensive investigation^7,8^, genetical and optical approaches have only just enabled us to tackle information flow through long-range connectivity in the sensory cortex in vivo^9–13^.

Long-range reciprocal connectivity also links sensory and motor cortical areas, thus forming sensory–motor cortical circuits. These circuits have been extensively studied in rodent whisker systems on an anatomical and physiological level^11–19^. Rather than processing information independently (Supplementary Fig. 1a), sensory and motor areas are tightly coupled to process two fundamentally different signals: externally evoked sensory information and internally generated motor information. The notion of processing two different signals in a distributed network is supported by the existence of motor signals in sensory areas and sensory signals in motor areas. For instance, sensory stimulation causes depolarization in the motor cortex due to long-range connections between the somatosensory and motor cortices^14^. Similarly, connections from the motor cortex can enhance sensory responses in the somatosensory cortex^15^. This overlapping network of sensory and motor signals raises a fundamental question of what information is communicated between these two cortical areas. One possibility is that the connectivity operates as a recurrent loop in which similar information is sent back and forth in both directions (Supplementary Fig. 1b), analogous to signal amplification mechanisms proposed in recurrent local networks^20,21^. Another possibility is that conveyed information is direction dependent and streamlined; in this case, the sensory cortex would select only sensory information from intermixed signals to send to the motor cortex, and the motor cortex would select only motor information to convey back to the sensory cortex (Supplementary Fig. 1c). Such direction-dependent flow of information might contrast with the extensive interactions and similarities between sensory and motor cortices but could increase the computational capacity as has been proposed in generative models of hierarchical cortical processing^4,22^.

To distinguish between these two possibilities, we investigated sensory–motor network activity in mice undergoing a visually guided eye movement task. By employing this task, we identified a small region in the secondary motor cortex (MO_s_) that controls eye movements and found that this area was reciprocally connected to a rostrolateral part of the higher visual areas (V_RL/A/AL_). These sensory and motor areas encoded both sensory information and motor signals, implying that they operate as a distributed network. However, the connections from the sensory to the motor area primarily conveyed sensory information and those from the motor to the sensory area preferentially carried motor signals. Our results provide insight into how reciprocal connectivity operates in a distributed network of sensory–motor interactions.

## Results

### A visually guided eye movement task in mice

**Fig. 1:**
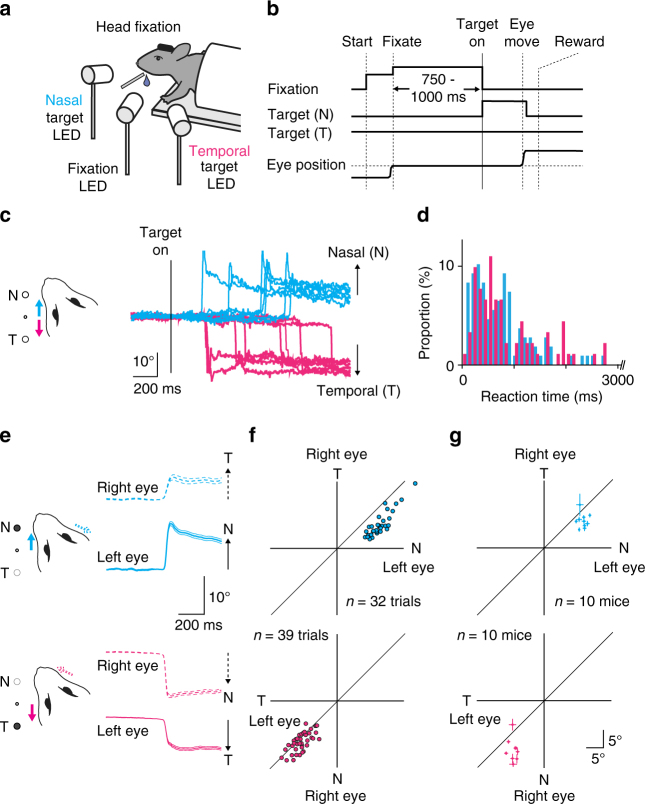
A visually guided eye movement task in mice. **a** The experimental design. Three LEDs instructed when and where to move the left eye. **b** After the mice fixated the central LED, one of the two LED targets (nasal or temporal) was turned on, and mice were required to shift their left eye toward the target. **c** Representative eye traces recorded during one behavioral session. Traces are aligned to the target onset. Cyan traces, trials with nasal target; magenta traces, trials with temporal target. **d** The distributions of reaction times for one animal (cyan, nasal target condition; magenta, temporal target condition). Reaction time range <3 s (nasal 91.7%; temporal 81.3%) is shown because of the long tail of its distributions. **e** Average traces for eye movement for both eyes in one behavioral session (nasal, 32 trials, cyan; temporal, 39 trials, magenta). Traces for the left eye, solid lines; right eye, dotted lines. Thick and thin lines represent mean ± s.e.m. **f** The amplitude of the eye movements for the left and right eyes (cyan, nasal target; magenta, temporal). The same animal as in **e**. **g** The average eye movement amplitude is shown for the nasal (cyan) or temporal (magenta) target trials in 10 mice. Error bars indicate s.e.m

Despite their significance in the visual system, visually guided eye movements have been little studied in mice. We developed a behavioral task in which mice first had to identify an external visual stimulus as sensory input and then generate eye movements as motor output. In this task, we trained head-fixed mice to direct their left eye toward a central fixation light-emitting diode (LED) (Fig. 1a, b and Supplementary Fig. 2). Within 750–1000 ms, we turned on one of the two target LEDs (nasal or temporal) to instruct the mice which direction they should move their eye. If the mice successfully shifted their left eye in the direction of the target LED, we rewarded them with a drop of water. After several weeks of training, mice became capable of performing eye shifts (Fig. 1c, d) with a success rate of 85.9 ± 2.2% and a reaction time of 772 ± 118 ms (*n* = 19 mice). During the task, the movement of other body parts, such as the forelimbs or whiskers, did not correlate with eye movements or the onset of the visual target (Supplementary Fig. 3).

Unexpectedly, even though the task only required movement of the left eye, both eyes moved together as a coupled movement (Fig. 1e and Supplementary Movie 1). When the left eye moved toward the nasal side (solid cyan trace in Fig. 1e), the right eye moved toward the temporal side (dotted cyan trace); when the left eye moved toward the temporal side (solid magenta), the right eye moved toward the nasal side (dotted magenta). The movements of the two eyes were highly synchronized, although the amplitudes and peak velocities were smaller for temporal than nasal eye movements (amplitude: 13.5 ± 0.8 vs. 8.8 ± 0.4 degrees; velocity: 1019 ± 46 vs. 498 ± 23 degrees/s; Fig. 1f, g, Supplementary Fig. 4; both eyes filmed for 10 mice).

The eye movements that we observed were qualitatively similar to saccades in primates albeit with some notable differences. First, the reaction time (772 ± 118 ms) was longer compared to that in macaques^23^ and humans^24^ (~200 ms). Second, the peak velocity of the eye movements was larger than that of macaque^25^ and human^26^ saccades with similar amplitudes (~300 degrees/s for 10 degrees saccades). Third, the post-eye movement drifts were larger than in primates^27^. Finally, in mice the nasal eye movements were larger than the corresponding temporal eye movements, but the reverse is true in humans^28^. Despite these differences, the binocular coupling of voluntary eye movements suggests that the mouse brain implements basic circuits for binocular eye movements, similar to those in foveate mammals.

### Secondary motor cortex is required for eye movement task

We investigated whether the motor cortex was required for the eye movements during our behavioral task by performing optogenetic suppression. As a first step toward optogenetic suppression, we identified a candidate region involved in eye movement. We performed systematic microstimulation in a medial portion of the frontal cortex. Low-current microstimulation (up to 50 µA) in awake mice evoked binocularly coupled rapid eye shifts (Supplementary Fig. 5a, b) when the stimulation was in a caudomedial portion of the frontal cortex (~700 µm depth; ~0.7 mm rostral and ~0.7 mm lateral to the bregma; Supplementary Fig. 5c–e). The same stimulation current could also evoke whisker movements in a wide region of the frontal cortex as previously reported^17^. A much stronger current (up to 200 µA) could elicit eye movements reliably even in other areas, such as the anterior cingulate area^29^ (data not shown). In subsequent experiments using optogenetics and imaging, we targeted the coordinate ~0.7 mm lateral and ~0.7 mm rostral to the bregma; this part of frontal cortex was free of large vessels, reliably elicited eye movements as large as those seen during the task (Supplementary Fig. 5e), anatomically projected to eye-movement related areas of the midbrain (Supplementary Fig. 6), and was located within the secondary motor cortex, a broadly defined area in the frontal cortex^7^ (Supplementary Fig. 5c–o). In this study, the region at this coordinate is referred to as the MO_s_.

**Fig. 2:**
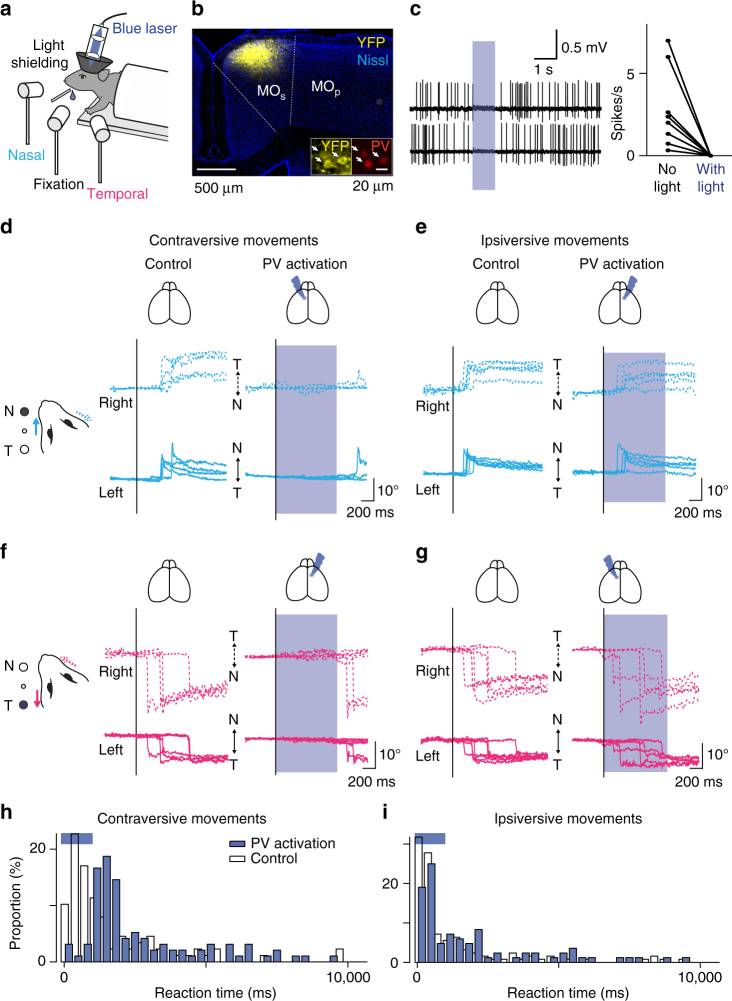
Optogenetic suppression of the MO_s_ during the visually guided eye movement task. **a** The experimental design. **b** ChR2 was expressed virally in PV interneurons in *Pvalb*-IRES-Cre transgenic mice. AAV-DIO-ChR2-eYFP was injected into the MO_s_ (coronal section stained with Nissl), and ChR2-eYFP expression was confined to cells expressing PV (white arrows). **c** Firing was suppressed during blue light illumination. Left, two representative neurons are shown. Right, blue light illumination completely suppressed firing activity in all eight neurons (*p* < 0.01, Wilcoxon signed-rank test). The activity was monitored with blind cell-attached recording. **d**–**g** Optogenetic suppression of the MO_s_ during the task. The virus was injected into the MO_s_ either in the left (**d**, **g**) or right (**e**, **f**) hemisphere. Unilateral suppression of the MO_s_ severely impaired eye movements in the contraversive (**d**, **f**) but only mildly in the ipsiversive (**e**, **g**) direction. **h**, **i** Reaction times for the eye movements. PV activation delayed the onset of contraversive eye movements (**h**, PV activation, *n* = 96 trials; control, *n* = 88 trials; *p* < 10^−11^), but showed only minor effects on ipsiversive movements (**i**, PV activation, *n* = 84 trial; control, *n* = 126 trials; *p* = 0.0501, Person’s chi-square test)

To examine whether the MO_s_ is necessary for voluntary eye movements, we optogenetically suppressed neural activity in this region during the eye movement task. Specifically, we locally activated parvalbumin (PV) interneurons that expressed channelrhodopsin-2 (ChR2) via viral transduction^30,31^ (Fig. 2a–c). The optogenetic suppression impeded contraversive movements severely (Fig. 2d, f, h, PV activation, *n* = 96 trials; control, *n* = 88 trials; *p* < 10^−11^, Pearson’s chi-square test) but had only minor effects on ipsiversive movements (i.e., toward the ipsilateral side, PV activation, *n* = 84 trial; control, *n* = 126 trials; *p* = 0.0501, Fig. 2e, g, i). In contrast, optogenetic suppression of the primary motor cortex, ~1 mm away from MO_s_, did not affect eye movements (Supplementary Fig. 7). These results suggest that the MO_s_ but not primary motor cortex is critical for visually guided eye movements, particularly contraversive ones.

### The MO_s_ encodes both motor signals and visual information

**Fig. 3:**
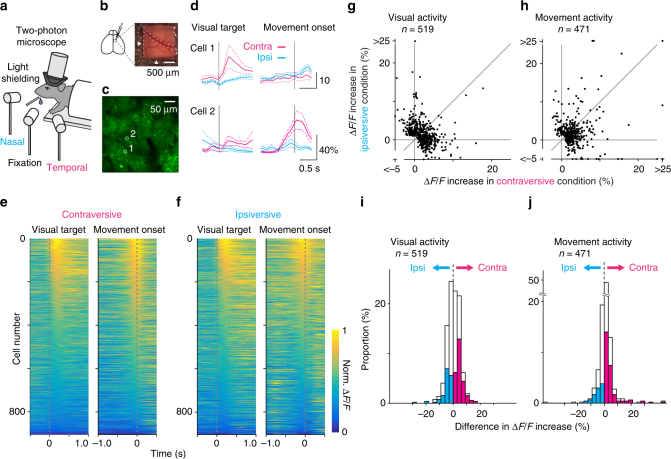
Neural coding in the MO_s_. **a** The experimental design. **b** Cranial window for two-photon imaging. Arrowheads indicate 700 µm rostral and 700 µm lateral from the bregma. Note a bridging vein leading to the superior sagittal sinus, which often runs around the coordinates. **c** Virally expressed GCaMP6f in the MO_s_ for two-photon calcium imaging during the visually guided eye movement task. **d** Averaged fluorescence change of two example cells (ROIs shown in **c**, aligned to the onset of the visual target or to the onset of the eye movement; cyan, the trial condition for ipsiversive eye movement; magenta, for contraversive). **e**, **f** Sorted normalized ∆*F*/*F* traces for 904 neurons showing significant response either to visual targets or to eye movements. Response was aligned to the onset of the visual target or the movement in the contraversive (**e**) or ipsiversive (**f**) trials. **g**–**j** Comparison between contraversive and ipsiversive movement trials for visual and motor activity (*n* = 519 or 471 neurons, showing significant visual or motor activity). **i**, **j** A histogram for difference between ipsiversive and contraversive conditions in visual or motor activity. Neurons with a larger response in the contraversive condition (*p* < 0.05, Mann–Whitney test) were labeled as magenta (*n* = 135 in visual activity, 122 in motor activity) and neurons with a larger response in the ipsiversive condition in cyan (*n* = 80, 45). The population preferred for the contraversive condition in visual activity (*n* = 519, *p* < 0.05, Wilcoxon signed-rank test) and in motor activity (*n* = 471, *p* < 10^−12^)

We predicted that the cortical area that controls eye movements would show neural activity related to the motor commands. To confirm this prediction, we investigated the neural activity of layer 2/3 neurons in the MO_s_ of head-fixed mice while they performed the eye movement task (Fig. 3a). We imaged fluorescent signals of the virally expressed, genetically encoded calcium indicator GCaMP6f as a readout for neural activity^32^ (Fig. 3b–d, Supplementary Fig. 5o). To help separate motor signals from the visual response, we rewarded mice for performing eye movements with a longer reaction time (Supplementary Fig. 8) and analyzed only trials with longer reaction times ( > 1.5 s). Twelve percent of neurons (471 out of 3787) showed an increase in fluorescent signals prior to the onset of eye movements (Fig. 3d–f). Previous studies in macaques considered neural activity preceding the onset of the eye movements to represent motor commands^23,33–35^; therefore, we will refer to this neural activity as motor activity. In contrast, neural activity after the onset of eye movements is unlikely to represent the motor command, and rather may be a mixture of visual activity, residual motor activity, and post-movement motor-related activity^36^. Across the population of neurons, motor activity showed a preference for the contraversive movement condition (Fig. 3h, j, *n* = 471, *p* < 10^−12^, Wilcoxon signed-rank test), consistent with the role of the MO_s_ in contraversive eye movements. The preference started 250 ms prior to the onset (Supplementary Fig. 9f–j).

The MO_s_ also encoded visual information to instruct the eye movements. Fourteen percent of neurons (519 out of 3787) showed an increase in fluorescence signal immediately after the presentation of a visual target (Fig. 3d–f). Previous studies reported visual activity in this region just after visual stimulation in anesthetized or awake quiet mice^37,38^; therefore, we will refer to this activity as visual activity. Consistently, we found that this activity was not significantly affected by the presence or absence of eye movements (Supplementary Fig. 10) and showed a slight preference for contraversive movement (Fig. 3g, i; *n* = 519, *p* < 0.05, Wilcoxon signed-rank test). The preference was relatively stable at least up to 800 ms after the onset (Supplementary Fig. 9a-e). Taken together, our in vivo calcium imaging experiments demonstrate that the neurons in the MO_s_ represent visual information immediately after the visual target and motor signals just before movement onset.

### The MO_s_ is reciprocally connected with visual areas RL/A/AL

**Fig. 4:**
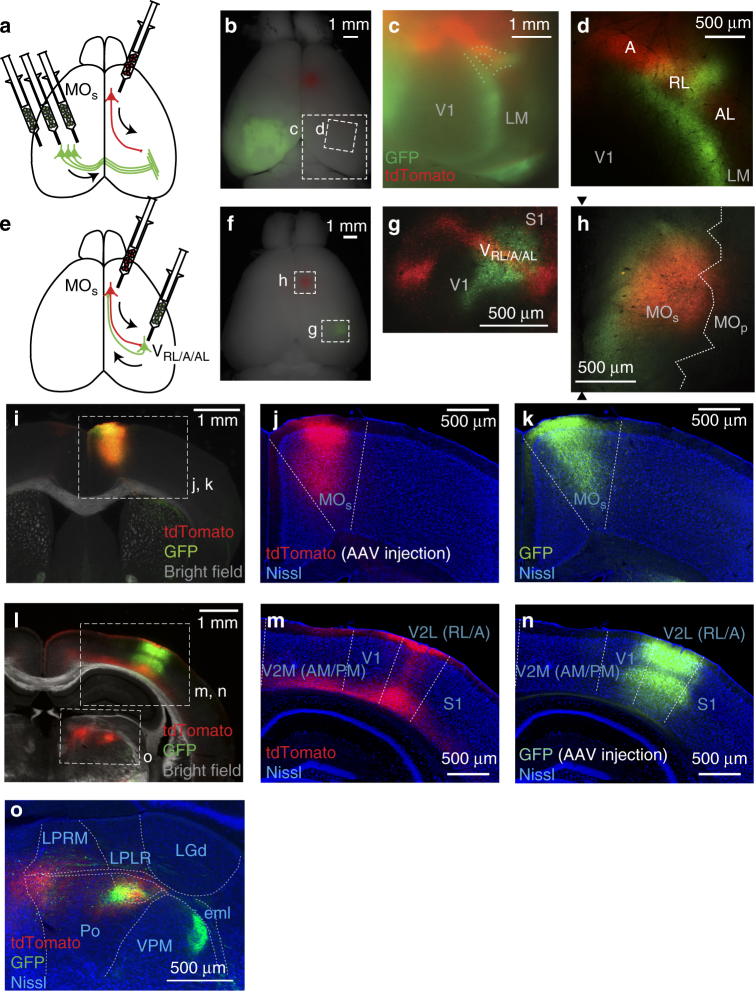
Reciprocal connectivity between MO_s_ and V_RL/A/AL_. **a**–**d** Connectivity from the MO_s_ to the V_RL/A/AL_. **a** The experimental design for the dual-virus injections. AAV-tdTomato was injected into the MO_s_ in the right hemisphere and AAV-GFP was injected into the occipital regions in the left hemisphere. **b** A corresponding whole-brain image. **c**, **d** The tdTomato projection from MO_s_ overlapped with areas RL, A, and AL (V_RL/A/AL_). GFP-labeled callosal projections delineated boundaries between the V1 and higher visual areas. The image **c** was taken with a fluorescent stereoscopic microscope from the dotted rectangle in **b**, and the image **d** was taken with a two-photon microscope from a region indicated in **b**. **e**–**n** The V_RL/A/AL_ projects back to the MO_s_. **e** The experimental design for the sequential virus injections. First, AAV-tdTomato was injected into the MOs, and then a couple weeks later AAV-GFP was injected into the tdTomato-labeled axonal band (the V_RL/A/AL_). **f** A corresponding whole-brain image. **g** At the injection site of AAV-GFP, the expression overlapped with the tdTomato-labeled axonal band (the location of V_RL/A/AL_). **h** At the injection site of AAV-tdTomato (the MO_s_), the expression overlapped with the GFP-labeled axonal band. The two black arrowheads indicate the midline, and the white dotted line is the expected areal border based on coordinates and Paxinos and Franklin’s atlas. **i**–**k** A coronal section that includes the MO_s_. The injection site for AAV-tdTomato at MO_s_ (**j**, tdTomato) and the axon terminals from the V_RL/A/AL_ (**k**, GFP) overlapped well. **l**–**n** A coronal section that includes V_RL/A/AL_. The axon terminals originating from the MO_s_ (**m**, tdTomato) and the inection site for AAV-GFP (**n**, GFP) overlapped well. The dashed lines in **j**, **k**, **m**, and **n** indicate the areal borders based on Nissl staining. **o** Projections from the MO_s_ and the V_RL/A/AL_ in thalamus. Note that projections from the V_RL/A/AL_ to the higher visual thalamus (LPLR)^73^ overlapped with those from the MO_s_. The borders were drawn based on descriptions in refs. ^42, 74^

The encoding of visual information in the MO_s_ implies that the MO_s_ interacts extensively with the visual cortex through long-range anatomical connections. Indeed, by labeling axon terminals of the MO_s_ with tdTomato (Fig. 4), we found that the MO_s_ projects to parieto-occipital regions of the cortex, including the visual cortex (~2.0 mm posterior and ~2.5 mm lateral to the bregma). To identify to where in the visual cortex the MO_s_ projects, we located the visual areas based on callosal connectivity as a landmark^39–41^; the callosal terminals expressing green fluorescent protein (GFP) visualized the boundaries among the primary visual cortex (V1) and higher visual areas in vivo (Fig. 4a–d). The MO_s_ projections formed a prominent band of tdTomato fluorescence, the lateral part of which was located rostrolateral to the GFP-labeled boundaries between the V1 and higher visual areas. In particular, the band of MO_s_ projections overlapped with a ring structure of callosal connectivity, which represented area RL (ref. ^39^, also known as the ‘rostrolateral visual area’ in the nomenclature of the Allen Reference atlas^7^; Fig. 4c, d). In addition to area RL, the band appeared to coincide with areas A (‘anterior area’) and AL (‘anterolateral’) in the visual cortex and to a small degree with the V1. We confirmed this pattern of MO_s_ projections with Nissl-stained coronal sections (Fig. 4j, m) where areas RL, A, and AL belong to a single cytoarchitectural region, the lateral area of the secondary visual cortex (V2L, the nomenclature of Paxinos and Franklin’s atlas^42^). Again, the MO_s_ projections coincided weakly with the V1 but well with V2L (Fig. 4m). These rostrolateral higher visual areas likely receive information related to eye movements from the MO_s_, consistent with the hypothesis that the RL, A, and AL areas fit in an anatomical diagram that resembles the dorsal visual stream^41^ specialized for visuospatial processing and guiding actions^43^. However, unlike the MO_s_, electrical stimulation in these areas rarely evoked eye movements (Supplementary Fig. 11) unless the stimulation current was doubled or tripled (up to over 200 µA), supporting the notion that these are mainly sensory areas. This study refers to these rostrolateral higher visual areas receiving MO_s_ projections as the V_RL/A/AL_.

A previous study reported projections from the RL, A, and AL areas to the secondary motor area^41^, suggesting that the long-range connections between the MO_s_ and the V_RL/A/AL_ are reciprocal. To confirm the reciprocal connectivity, we conducted sequential virus injections (Fig. 4e). We delivered the first virus (AAV-tdTomato) to the MO_s_ (Fig. 4f, h, j) to visualize its projection band, representing the V_RL/A/AL_. A few weeks later, we injected a second virus (AAV-GFP, green signal in Fig. 4g, n) into the middle of the tdTomato band (red signal in Fig. 4g, m, expected to be around the RL) to label the projections from the V_RL/A/AL_. Through this sequential approach, we found that GFP-labeled projections from the V_RL/A/AL_ overlapped with the first injection site (AAV-tdTomato) in the MO_s_ (Fig. 4h, i). Therefore, we conclude that the MO_s_ interacts with the V_RL/A/AL_ through long-range reciprocal connectivity.

We further confirmed the strong reciprocal connections between the MO_s_ and the V_RL/A/AL_ with a database available from the Allen Institute for Brain Science^7^. Virus injected into coordinates 500–1000 mm rostral and lateral to the bregma (designated as the secondary motor area in their nomenclature) labeled a prominent axonal band similar to ours, which covered areas RL, A, and AL (Supplementary Fig. 12a–c). The axonal density in these areas was 14.0 ± 4.2 times higher than in the V1 (*n* = 7, Supplementary Fig. 12f). Again similar to our results, virus injection into area RL/A/AL labeled axon terminals densely at coordinates ~700 mm rostral and lateral to the bregma in the secondary motor cortex (Supplementary Fig. 12h–j, m). Injection into the V1 labeled more selectively in the anterior cingulate area^44^ (Supplementary Fig. 12n). The database supports that the MO_s_ and the V_RL/A/AL_ are strongly linked with each other. In addition, the database confirms the parallel reciprocal pathways reported in the frontal-parieto-occipital cortex^44,45^; the secondary motor area ~0.5 mm rostrolateral to our injection site is reciprocally linked with the primary somatosensory cortex (Supplementary Fig. 12e, l), and the dorsal part of the anterior cingulate area is linked with the V1 (Supplementary Fig. 12g, n). However, these three sets of reciprocal connections overlapped with each other, indicating that the connectivity was diffuse. Altogether, the database illustrates reciprocal connectivity in frontal-parieto-occipital cortex, including the MO_s_ and the V_RL/A/AL_.

### Mixed encoding of visual/motor signal in V_RL/A/AL_ but not in V1

**Fig. 5:**
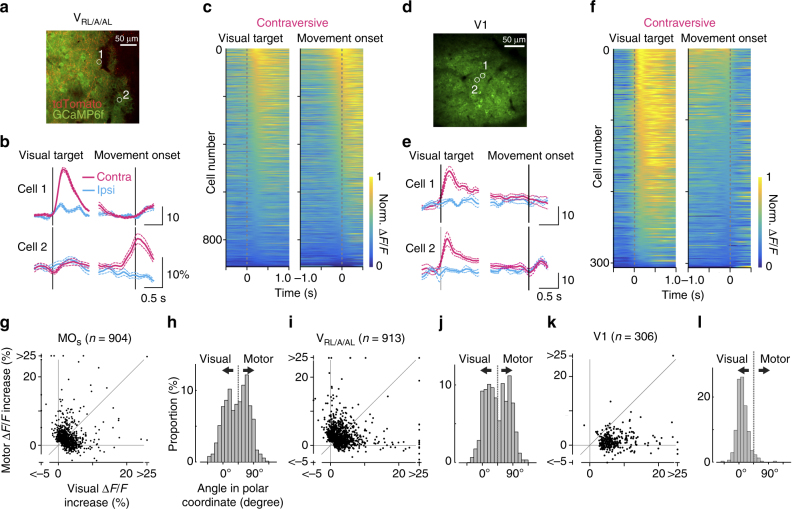
Neural coding in the V_RL/A/AL_ and the V1. **a**–**c** Coding in the V_RL/A/AL_. **a** GCaMP6f expression at layer 2/3 in the V_RL/A/AL_. The position of the V_RL/A/AL_ was determined with fluorescent guidance of tdTomato-labeled projections from the MO_s_. **b** Representative cells. Corresponding ROIs are shown in **a**. **c** Sorted normalized ∆*F*/*F* responses for neurons showing significant response (*n* = 913). Traces were aligned to either the onset of the visual target or the movement in the contraversive movement condition. **d**–**f** Coding in the V1. **d** GCaMP6f expression at layer 2/3 in the V1. **e** Representative cells. **f** Sorted normalized ∆*F*/*F* responses for neurons with a significant response (*n* = 306). **g**–**l** Comparison between visual and motor activity for neurons in the MO_s_ (**g**, **h**, *n* = 904), V_RL/A/AL_ (**i**, **j** *n* = 913), and V1 (**k**, **l**, *n* = 306). **g**, **i**, **k** Visual and motor activity was plotted for each neuron. **h**, **j**, **l** The angle in polar coordinates was computed for each neuron and plotted as a histogram. Degrees of 0 or 90 indicate pure visual or motor activity

The strong reciprocal connections between the V_RL/A/AL_ and the MO_s_ raised the possibility that neural activity in the V_RL/A/AL_ is similar to that in the MO_s_. Therefore, we investigated neural activity in the V_RL/A/AL_ by measuring fluorescence changes of virally expressed GCaMP6f from layer 2/3 neurons. Neurons in the V_RL/A/AL_, which we identified by labeling the MO_s_ projection terminals with tdTomato (Fig. 5a, red signal, Supplementary Fig. 13a), showed a visual response and motor activity, similar to the MO_s_ (visual activity, 23% of 2321 neurons; motor activity, 20%, Fig. 5b, c). The V_RL/A/AL_ also had more neurons that preferred contraversive movement (Supplementary Fig. 14c, d). In contrast, the V1, which was only weakly connected with the MO_s_, showed little motor activity (visual activity, 57% of 531 neurons; motor activity, 0.9% of 531 neurons; Fig. 5d–f). To evaluate the preference for visual or motor activity in the V1, MO_s_, and V_RL/A/AL_, we computed the polar angle for individual neurons based on the Cartesian representation of visual and motor activity (Fig. 5g, i, k). In the polar representation, the angle of 90° indicates strong preference for motor information, and 0° for visual information. The distribution of the polar angles indicate that the preference for motor over visual activity across neurons in the V_RL/A/AL_ was significantly different from the V1 but not the MO_s_ (Fig. 5h, j, l, MO_s_, *n* = 904, V_RL/A/AL_, *n* = 913, V1, *n* = 306; *p* < 10^−43^ and *p* = 0.06, respectively, Mann–Whitney test). Preference for motor activity over post-movement activity was similarly evaluated and was found to be slightly less in the V_RL/A/AL_ than the MO_s_ (Supplementary Fig. 15b, d, MO_s_, *n* = 669, V_RL/A/AL_, *n* = 705; *p* < 0.001, Mann–Whitney test) but much stronger than the V1 (Supplementary Fig. 15d, f, V_RL/A/AL_, *n* = 705; V1, *n* = 54; *p* < 10^−9^, Mann–Whitney test, the post-movement activity represents an intricate mixture of visual activity, remaining motor signals, and post-movement motor-related activity^36^). These results indicate that the MO_s_ and the V_RL/A/AL_ encode qualitatively similar patterns of visual and motor activity and suggest that the reciprocally connected sensory and motor areas form a distributed network in sensory–motor processing.

### Direction-dependent flow of sensory–motor information

**Fig. 6:**
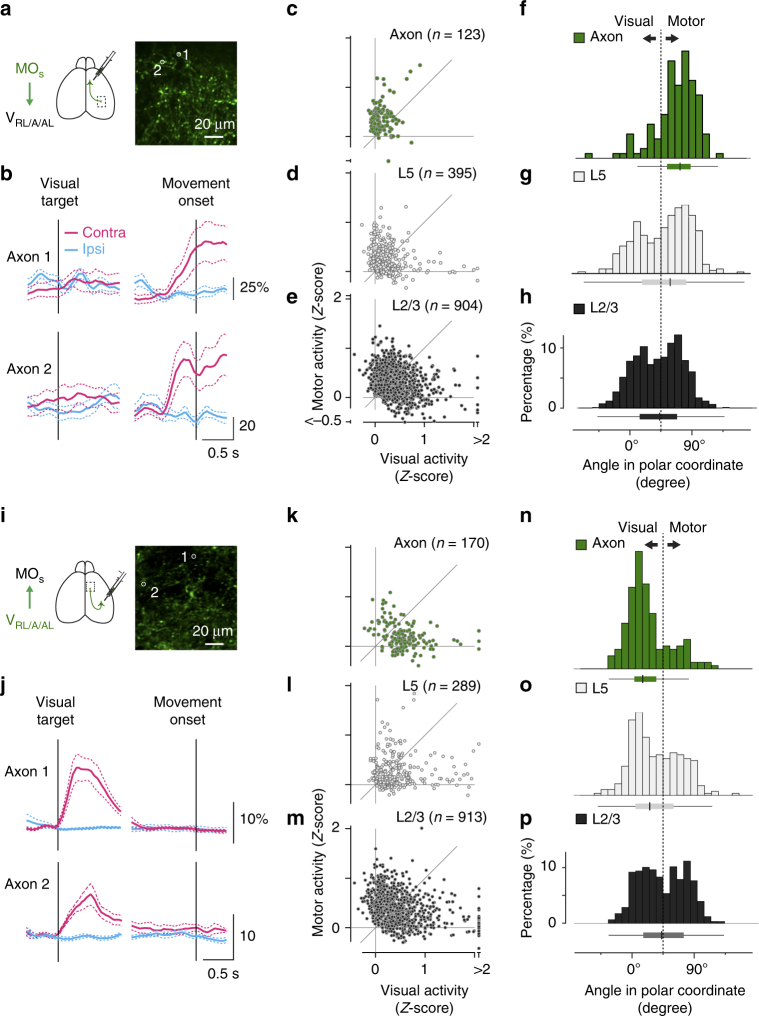
Biased neural coding in MO_s_ and V_RL/A/AL_ axons. **a**–**c** Coding in MO_s_ axon terminals projecting to the V_RL/A/AL_. **a** GCaMP6f was expressed in MO_s_ neurons, and their axons were imaged in the V_RL/A/AL_. **b** Calcium traces from two representative axons (mean ± s.e.m., cyan for the ipsiversive movement condition; magenta for the contraversive). The corresponding ROIs are shown in **a**. **c**–**e** Comparison between visual and motor activity for MO_s_ axons (**c**, *n* = 123), L5 somas (**d**, *n* = 395), and L2/3 somas (**e**, *n* = 904). **f**–**h** Histograms of the angle in polar coordinates for MO_s_ axons, L5 neurons, and L2/3 neurons. The distribution of the axons is more biased towards 90 degrees, indicating higher motor selectivity. Median, 25% quantile, and 75% quantile are represented as box plots, whisker length is 1.5 of the interquartile range. **i**–**p** Similar to **a**–**h**, but for V_RL/A/AL_ axon terminals projecting to the MO_s_. V_RL/A/AL_ axons, *n* = 170; V_RL/A/AL_ L5 somas, *n* = 289; L2/3 somas, *n* = 913. Note skewed distribution of the axons towards 0 degrees, indicating higher visual selectivity. The histograms for L2/3 somas (**h**, **p**) are the same with those in Fig. 5h, j

The similar encoding patterns in the MO_s_ and the V_RL/A/AL_ raise the question of how these two areas communicate with each other in a distributed network. One possibility is that the connections between these two areas underlie reverberant neural activity; the reciprocal connections might carry similar information in both directions (Supplementary Fig. 1b). Alternatively, the connections between them might preferentially convey specific types of information depending on the direction of the connectivity, indicating streamlined information flow in the distributed network (Supplementary Fig. 1c). To distinguish between these possibilities, we imaged calcium signals of projected axon terminals^18^ in two directions: from the MO_s_ to the V_RL/A/AL_ (Fig. 6a, b) and from the V_RL/A/AL_ to the MO_s_ (Fig. 6i, j, Supplementary Fig. 13b). Compared to layer 2/3 neurons in the MO_s_, axon terminals from the MO_s_ to the V_RL/A/AL_ showed a polar angle more distributed around 90° (Fig. 6c, f), exhibiting more motor activity than visual activity (*p* < 10^−14^, Mann–Whitney test, 123 axon terminals vs. 904 layer 2/3 somas, Fig. 6f, h). Higher selectivity for motor information in axon terminals indicates that the MO_s_ favorably conveys motor signals to the V_RL/A/AL_. In contrast, axon terminals from the V_RL/A/AL_ to the MO_s_ (Fig. 6k, n) showed a stronger preference for visual activity compared to layer 2/3 neurons in the V_RL/A/AL_ (*p* < 10^−13^, 170 axon terminals vs. 913 layer 2/3 somas, Fig. 6n, p). In addition to layer 2/3 neurons, another major source of long-range axonal projections is layer 5a neurons, which showed higher selectivity than layer 2/3 neurons in the MO_s_ (*p* < 10^−4^, Mann–Whitney test, 395 layer 5a somas: Fig. 6d, g, 904 layer 2/3 somas: Fig. 6e, h) and in the V_RL/A/AL_ (*p* < 10^−5^, Mann–Whitney test, 289 layer 5a somas: Fig. 6l, o, 913 layer 2/3 somas: Fig. 6m, p). Nonetheless, we observed even higher selectivity in axon terminals than in layer 5a neurons in both the MO_s_ (*p* < 10^−4^, Mann–Whitney test, 123 axon terminals vs. 395 layer 5a somas; Fig. 6f, g) and the V_RL/A/AL_ (*p* < 10^−3^, Mann–Whitney test, 170 axon terminals vs. 289 layer 5a somas; Fig. 6n, o). This direction-dependent information flow between the MO_s_ and the V_RL/A/AL_ suggests that the reciprocal connectivity acts as a bidirectional filter in the distributed network.

## Discussion

The cerebral cortex consists of intricate networks of long-range connections, many of which are reciprocal. Such reciprocal connectivity is particularly prominent between sensory and motor cortical areas, and yet how the networks operate in vivo through the connectivity has been little investigated. In this study, we discovered that communication through reciprocal connections between the MO_s_ and the V_RL/A/AL_ depends on the direction of the connectivity, suggesting that they function as a bidirectional filter. Thus our findings provide insight into how reciprocal long-range connectivity contributes to communication within a distributed network of cortical areas.

Our study showed that mice possess basic cortical circuits for eye movement, in addition to the circuits for the visual system that have been investigated intensively^46^. Although eye movements have been reported in awake rodents head-fixed at rest^47–49^ or running^50^ and in those freely moving^51^, it was unclear whether those movements were produced voluntarily. Our approach, which incorporated an eye movement task, optogenetic suppression, and in vivo two-photon imaging, provided converging evidence that mice are capable of shifting their eyes voluntarily. Furthermore, we found that these binocularly coupled eye movements were controlled by a small portion of the frontal cortex, located within the secondary motor cortex. It was unexpected that this area controls binocularly coupled eye movements, as mice have afoveate retinas, a limited binocular visual field due to their lateralized eyes, and independent control over their two eyes by vestibular inputs^51^. One possible explanation for these binocularly coupled eye movements is that they might play a role in maintaining binocular visual fusion. Although the binocular coupled eye movements in mice were qualitatively similar to those in primates, and the projection patterns of the mouse MO_s_ to higher visual areas were reminiscent of those of the primate frontal eye field (FEF)^52^, variable amplitude of eye movements at the same electrical stimulation current (Supplementary Fig. 5a), together with the lack of distinct cytoarchitecture^42^, contrasts with the primate FEF. Therefore, it is premature to conclude that this portion of the secondary motor cortex is specialized for eye movements or is a primitive homolog of primate cortical areas for eye movements (i.e., the FEF (ref. ^33^), the supplemental eye field (SEF)^35)^, and the rat frontal orienting field (FOF)^53^. Future studies will be needed to determine whether this MO_s_ portion contains the same highly sophisticated functions and anatomical connectivity as the primate FEF, SEF, and the rat FOF.

Our visually guided eye movement task provided us with a unique opportunity to investigate how motor activity is represented in the visual cortex, which consists of the V1 and higher visual areas. These visual areas are known to have their own preferences for particular visual stimuli^5,6^ and our finding also shows their distinct preferences for eye motor signals, prominent in higher areas of the dorsal stream^41^ (i.e., the V_RL/A/AL_) but little in the V1. Similarly, the V_RL/A/AL_ showed a stronger preference for eye motor signals over post-movement activity than the V1, although this post-movement activity is not straightforward to interpret because of the intricate mixture of visual activity, remaining motor signal, and post-movement motor-related activity^36^. These distinct preferences for eye motor signals coincided with anatomical projections from the MO_s_ to the visual cortex; the MO_s_ sent strong projections not to the V1 but to the V_RL/A/AL_. Therefore, motor signals in visual areas might be shaped by projections from the MO_s_, which is more biased to motor information.

We found that the transfer of neural information between the MO_s_ and the V_RL/A/AL_ depended on the direction of the long-range connections. Previous studies have investigated how long-range connectivity carries information in the outgoing directions: how the primary somatosensory area sorts and conveys specific information to the secondary somatosensory area and to the motor cortex^11–13^. However, whether and how long-range connections convey specific information in different directions in reciprocal connectivity was unclear. A recent study suggested the intriguing possibility that the reciprocal connectivity between the primary somatosensory area and the secondary motor area serves as a recurrent loop to generate reverberant activity^54^. Our study reveals another possibility that the reciprocal connectivity in the visuo-oculomotor cortical circuit involving the V_RL/A/AL_ preferentially conveys different types of information depending on the direction of information flow. Future research will be required to understand the physiological role of this reciprocal connectivity. For instance, it would be interesting to determine whether the selected information is used in the motor area for visuomotor transformation^55^ and in the visual areas for remapping of visual space and visuospatial attention^56^ as in primates.

What are the computational advantages to streamlining the information flow between the reciprocally connected areas of mixed sensory and motor representations? Streamlined flow might indicate the existence of processing units that are functionally localized even in a distributed network. Another possibility is that streamlined inflow in a distributed network may drive mixed selectivity; streamlined inflow from separate input sources might easily achieve mixed selectivity, which is known to be computationally powerful^57–59^. It will be interesting to investigate which of these possibilities, streamlined flow or mixed selectivity, is more strongly linked to behavioral performance^57^. Our findings may provide a foundation for investigating the computational power of reciprocal connectivity, which is abundant in the cortex.

## Methods

### Mice

All experimental procedures were approved by the University of Tübingen, National Center of Neurology and Psychiatry, and local institutions in charge of animal experiments. C57BL/6J and PV–Cre mice (JAX stock #008069)^60^ were used in this study, including 10 mice for binocular eye movement analysis, 11 for microstimulation, 17 for optogenetic inhibition, 9 for anatomical tracing, and 35 for two-photon imaging (8 for imaging of MO_s_ layer 2/3, 6 for V_RL/A/AL_ layer 2/3, 2 for V1, seven for MO_s_ layer 5, 4 for V_RL/A/AL_ layer 5, 5 for axon terminals from MO_s_, and 3 for axon terminals from V_RL/A/AL_). All wild-type mice were randomly assigned to the experiments. For the optogenetic suppression experiment, Cre-positive mice were selected by genotyping and then assigned randomly to each of the groups. All mice were male and aged >8 weeks. The mice were group housed (up to five mice in a cage), and experiments were performed during the dark period of the 12 h light/12 h dark cycle.

All surgical procedures were carried out aseptically under anesthesia with 100 mg/kg ketamine and 8 mg/kg xylazine (intraperitoneally), supplemented with isoflurane for maintenance. Lidocaine (subcutaneously at the incision), atropine (0.3 mg/kg, intraperitoneally), caprofen (5 mg/kg, intraperitoneally), and dexamethasone (2 mg/kg, intraperitoneally) were applied to prevent pain and brain edema. After surgery, mice were allowed to recover for at least 3 days. No experimenter blinding was done.

### Cranial window surgery

A custom-made headpost was glued and cemented to the skull, and then a craniotomy (1–2 mm circle or rectangle) was performed over the MO_s_ (Fig. 3b, ~0.7 mm anterior and ~0.7 mm lateral from the bregma, which is the best-fit intersection between the midline and the coronal suture), the V_RL/A/AL_ (2.0 mm posterior and 2.5 mm lateral from the bregma), or the V1 (0.0 mm posterior and 3.0 mm lateral from the lambda, which is the best-fit intersection between the midline and the lambdoid suture). The location of the V_RL/A/AL_ was guided by the axon terminals of the MO_s_ (see below). Inside the area of the craniotomy (above the MO_s_, V_RL/A/AL_, or V1), virus (AAV2/1-syn-GCaMP6f)^32^ was injected at a couple of sites (40–60 nl/site; depth, 200–300 μm; 3–5 min/injection), which resulted in virus expression of ~400 μm. An imaging window consisting of two or three layers of coverglass (a small trimmed glass piece glued onto a larger piece) was fixed to the skull with dental acrylic^61^ (Fig. 3b).

For axon terminal imaging, virus injection and imaging were performed in two different cortical areas (the MO_s_ and V_RL/A/AL_). A small hole (250 μm) was made in the bone over the MO_s_ (based on the coordinates) or V_RL/A/AL_ (with the guide of callosal connectivity, see below). Through this hole, virus (AAV2/1-syn-GCaMP6f)^32^ was injected at a depth of 200–300 μm. After virus injection, a headpost was implanted and a larger craniotomy (1–2 mm, circle or rectangle) was made above the other area (the V_RL/A/AL_ or MO_s_). Following craniotomy, a cranial glass window was implanted as described above. After virus injection, mice were allowed to recover in their home cages for a few days.

The position of the V_RL/A/AL_ for GCaMP6f virus injection was identified based on axonal projections from the MO_s_ or from the contralateral visual cortex (Supplementary Fig. 13). For somatic GCaMP6f imaging in the V_RL/A/AL_, the injection location was determined with the guide of axon terminals from the MO_s_ labeled beforehand with AAV2/1-EF1α-tdTomato or dextran conjugated with Alexa 594 (10% v/w in cortex buffer, total of 400 nl, Supplementary Fig. 13a). The MO_s_ projections label areas RL and A heavily, thereby our injections were mainly in these areas. For imaging axon terminals from the V_RL/A/AL_ to the MO_s_, GCaMP6f virus was injected into area RL and A with a guide of the callosal ring visualized by injecting AAV2/1-EF1α-tdTomato or dextran Alexa 594 into the contralataral visual cortex beforehand (Supplementary Fig. 13b).

### Behavioral training

Mice were pretrained to enter a tube to obtain a water reward (for 7–10 days). Over the last 2 days of pretraining, mice were acclimated to the imaging setup, and rapid eye movements were reinforced with a water reward. This reinforcement was crucial, as spontaneous eye movements became rare once the mice became used to the task environment. Following this pretraining, the mice were trained to perform a visually guided eye movement task. In this behavioral paradigm, three blue LEDs were used as visual stimuli: a central fixation LED, a nasal target LED, and a temporal target LED (wavelength 470 nm, M470F1, Thorlabs) (Supplementary Fig. 2). The fixation LED was placed 12 cm away from the left eye at an elevation angle of 20° and an azimuth angle of 60°. The nasal and temporal target LEDs were placed at an elevation angle of 20° and an azimuth angle of 35° and 85°, respectively. In front of each LED, a 475-nm short-pass filter (#84–692, OD4, Edmund) was placed to prevent stray light into the photomultiplier tube. A dichroic mirror (#43–958, Edmund) was placed between the LED and the eye, allowing light below 675 nm to be transmitted and causing light above 750 nm to be reflected (Supplementary Fig. 2a). This strategy enabled infrared (IR) light to monitor eye position without interference from the fixation or target LEDs. During each trial, the brightness of the LEDs indicated where the left eye should be located (Fig. 1b). The brightness was measured in μW with a power meter (OP-2 VIS, Coherent) at the output of the LED. The light of the fixation LED was first set at 260 μW for 3–4 s and then increased to 470 μW at the start of the trial. When the left eye of the mouse was directed to the fixation LED (within ± 2.5°), the brightness was further increased to 500 μW. The mouse was required to maintain the fixation for 750–1000 ms. If the mouse failed to do so, the fixation LED became dimmer (260 μW) for 1000 ms and then brighter again to indicate the beginning of the fixation period. The fixation break prior to the visual target onset was not considered an error. After successful fixation of 750–1000 ms, one of the two target LEDs (randomized) was turned on (450 μW), and at the same time, the brightness of the fixation LED was decreased (260 μW). The mouse was required to shift its gaze in the direction of the target LED within 10 s, which was sufficiently long to keep its motivation. Following successful eye movements, the target LED was turned off (70 ms later), and after a short delay of 200–300 ms, a drop of water was provided. The eye shift was required to be a rapid movement (amplitude >5°, speed >0.1°/ms). If the gaze moved out from the fixation window without the correct eye movement, the trial was considered as an error, an incorrect eye movement that can include a rapid movement in the wrong direction, a rapid movement at small amplitude, and a slow eye drift. The mouse generally performed this task ~50 times per day. For the imaging experiments, we encouraged mice to have longer reaction time by using a larger amount of reward for those trials (8–12 μl for reaction time shorter than 1 s, 20–25 μl for reaction time longer than 1 s up to 20 s). After doing this, the mice had a median reaction time of over 1 s (Supplementary Fig. 8); trials shorter than 1.5 s were excluded to facilitate separation of visual and motor activity.

The training procedure for this task was similar to that used for saccade tasks in primates^62,63^. Over the first several days, fixation to the central LED was encouraged with a small amount of water, and the task was made easier by having a large fixation window ( ± 5°). Upon successful eye movement, a larger reward (up to 50 μl) was provided. As the task performance improved, the reward amount was reduced (15–25 μl) and the size of the fixation window was decreased to  ± 2.5°, which rejects large eye drift and small eye movements. Some mice that stuck to eye drifts were excluded (*n* = 10 mice), as such drifts severely impeded improvement in behavioral performance. Behavior was monitored using a program written in TEMPO^62,63^. When we instructed the mice to move their eyes vertically, we could not keep them motivated as a consequence of too many consecutive errors. This was also the case when we turned off the target LED before the onset of eye movement (a memory-guided eye movement task).

### Measurement and analysis of eye position

The position of the left eye was monitored using a commercial camera package (Eyelink 1000, SR Research) sampled at 500 Hz during the task. The light source for the camera was a 940-nm LED. In front of the camera, an 800-nm long pass filter (#66–059, Edmund) and a 960-nm short pass filter (HQ960SP, Chroma) were placed to block the IR light for the right eye, blue LEDs, and two-photon laser. In some experiments, the image of the left eye was saved at 200 Hz with precise timing of individual frames, using a complementary metal-oxide semiconductor (CMOS) camera (DCC1240M, Thorlabs) that was controlled by custom-made software (written in Visual C++ with the Thorlabs Software Development Kit). The camera was placed at the same azimuth angle as the fixation LED. At the edge of the camera were four reference LEDs to confirm that the eye was directed toward the fixation LED^47^. Optical filters were similarly placed in front of the camera.

In some experiments, the position of the right eye was monitored using a second CMOS camera (DCC1240M, Thorlabs) with images saved at 200 Hz. The light source for this camera was a 780 nm LED (M780L2, Thorlabs). In front of the camera, a 960-nm short pass filter (HQ960SP, Chroma) was placed.

The position of the eye was determined for each frame using a custom-written program in MATLAB (Supplementary Fig. 2b). The image in each frame (Supplementary Fig. 2b1) was processed to produce two binary maps: (1) a threshold map based on discrimination analysis (Supplementary Fig. 2b2), identifying the light source and eyelids; and (2) a Canny map based on the Canny edge detector^64^ (Supplementary Fig. 2b3), detecting the boundary of the pupil, light source, and eyelids. By combining these two maps, the edges corresponding to the light source and those within the eyelids could be eliminated (Supplementary Fig. 2b4, a candidate map). The boundary of the pupil was determined by searching the best-fit circle with a circumference overlapping as many white pixels per length as possible in the candidate map. Once the pupil boundary was identified, the angular position of the eye was calculated using *x*, *y*, and *r*^47^.

### Microstimulation

Electrical microstimulation was conducted in awake mice to identify a candidate cortical region for eye movements in the frontal cortex (Supplementary Fig. 5). The mouse was head-fixed in the behavioral apparatus, and the positions of the two eyes were monitored. When the left eye was in its primary position (azimuth angle of ~60°), the stimulation was delivered using a tungsten or platinum/iridium electrode (amplitude, 20–50 µA; frequency, 200 Hz; pulse width, 300 μs; duration, 250 ms) at around layer 5, at a depth of 700 µm (or 1000–1500 µm for medial bank, region II in Supplementary Fig. 5c–e), and at 0.3–1.5 mm lateral to the bregma (or 0.4–0.7 mm for medial bank). The stimulation parameters were similar to those used in previous studies on motor regions in rodents^65^ and primates^34^. The effects of microstimulation were also examined at the location of the V_RL/A/AL_ and V1 (Supplementary Fig. 11). In most animals, the current value was fixed at the lowest value that could evoke reliable eye movements at 0.7 mm rostral and 0.7 mm lateral to the bregma, allowing many sites to be stimulated while the mice remained still. In two animals, we confirmed that a stronger current (~200 µA) could evoke eye movements in the anterior cingulate area, the V_RL/A/AL_, and superficial layers (at 300 µm) of the MO_s_.

In three experiments, we confirmed the electrode track in the MO_s_ with coronal sections. The electrode track was visualized by coating the electrode surface with DiO (Invitrogen, Carlsbad, CA, USA). The brain was perfused with 4% paraformaldehyde (PFA), cryoprotected, and then sectioned coronally with a cryotome (40 or 60 µm thickness). Sections were incubated in 0.1 M phosphate-buffered saline (PBS) containing Triton X-100 (Sigma-Aldrich, Buchs, Switzerland) and fluorescent Nissl (1:200, NeuroTrace, N-21482 or N-21483, Invitrogen) for Nissl staining. Sections were mounted on a slide glass and embedded in an anti-fade solution (Dako Fluorescence Mounting Medium, S3023, Agilent, Santa Clara, CA, USA). Areal borders were determined based on fluorescent Nissl signal and a standard atlas^42^. The fluorescent Nissl staining was also performed for virus-injected cortex (Supplementary Fig. 5o: N-21482 Invitrogen, Fig. 2b and Fig. 4: N-21483). The image was taken with an Axio Imager Z2 (Carl Zeiss Microscopy, Jena, Germany). The objective lens was 5× (NA = 0.13). The filter cube for DiO contained an excitation bandpass filter of 470/40, a dichroic filter of 495, and an emission bandpass filter of 525/50. The filter cube for red-fluorescent Nissl contained an excitation bandpass filter of 550/25, a dichroic filter of 570, and an emission bandpass filter of 605/70. The filter cube for deep-red fluorescent Nissl contained an excitation band filter of 640/30, a dichroic filter of 660, and an emission bandpass filter of 690/50.

### Three-dimensional reconstruction

In one experiment, we reconstructed 23 serial sections (60 µm thickness; every other section; ~2.6 mm) to depict the areal border of the secondary motor cortex in a microstimulated brain (Supplementary Figure 5f). First, we manually realigned each slice using MATLAB and then reconstructed serial sections with an Image J plug-in, 3D viewer. We similarly made a reconstruction for serial sections in Paxinos and Franklin’s atlas^42^. The dorsal view of the atlas was based on the projection of the 3D reconstruction (Supplementary Figure 5c–e, Fig. 4h). In the image of Fig. 4h, the anterior–posterior level of the injection site (0.7 mm anterior from the bregma) was estimated using Gaussian fitting of the tdTomato signal.

### Optogenetic inhibition

Optogenetic inhibition was accomplished by selectively activating PV+interneurons locally in PV-Cre mice (Fig. 2)^30,31^. Following headpost implantation and craniotomy, AAV2/1-EF1α-DIO-hChR2(H134R)-EYFP was injected into the MO_s_ unilaterally (4–6 sites; 60 nl/site; depth, 200–300 μm; expression ~ 1 mm) and a coverglass was placed (see “Cranial window surgery” section). A similar virus injection was performed 1 mm lateral to the MO_s_ for control experiments to inhibit the primary motor cortex. Photostimulation of ChR2 was achieved using a blue laser (473 nm, CrystaLaser) coupled to an optic fiber (M15L02, Thorlabs). The output power was controlled by combining a half-wave plate with a polarizing beamsplitter cube. An optical chopper was used to convert the continuous wave into a 40-Hz pulse^31^ (pulse width, 2.5 ms; Edmund optics, 59–894). The output of the optic fiber and the surface of the cortex were placed on conjugate planes using two convex lenses^40^ (5 cm focal lengths). A beam splitter was placed in the infinity space, and the reflected light was focused onto the sensor of a CMOS camera (Thorlabs). This design enabled monitoring the precise location of the stimulated site on the surface of the cortex. Optogenetic inhibition was applied in 25–60% of the trials, where a particular direction of eye movements was required. The direction of eye movements was randomized among mice. The average power of the light at the surface of the cortex was 600–1200 μW. The effect of optogenetic inhibition was quantified to compare the probability of eye movements within 1 s after the target onset between trials with and without the 1 s laser illumination (Person’s chi-square test).

### Cell-attached recording

Cell-attached recording was performed to confirm the effect of optogenetic inhibition (Fig. 2c). A small craniotomy was made (1 mm diameter), and then a patch pipette was advanced blindly while monitoring its impedance to achieve a loose-seal cell-attached recording. The recording pipette contained HEPES-based artificial cerebrospinal fluid. The signal was amplified with a patch clamp amplifier (MultiClamp 700B, Molecular Devices). Signals two standard deviations larger than baseline were considered spikes. Eight cells with broad spike width were recorded.

### Two-photon imaging

In vivo imaging was performed using a two-photon microscope based on a movable objective microscope system (Sutter) controlled by the ScanImage software^66^, as previously described^13,67^. The light source was a pulsed Ti:sapphire laser (Chameleon, Coherent), and the laser wavelength was set at 980 nm, which causes a high fluorescent change in GCaMP signal (“Two-Photon Fluorescent Probes”, https://www.janelia.org/lab/harris-lab-apig/research/photophysics/two-photon-fluorescent-probes) and less scattering in the tissue than shorter wavelengths. The objective lens was apochromatic (16×, 0.80 NA, Nikon). Red and green fluorescent photons were separated using a 565-nm dichroic mirror (Chroma, 565dcxr) and barrier filters (green, ET525/70 m-2p; red, ET605/70 m-2p). Signals were collected using photomultiplier tubes (Hamamatsu Photonics, H10770PA-40); frame scanning (frame rate 6–30 Hz) was used. Images were collected at a depth of 150–300 μm from the dural surface for layer 2/3 neurons, 75–100 μm for axonal boutons^10^, or 450–550 μm for layer 5a neurons, as previously described^67^. For layer 5 imaging, the back aperture of the objective was under-filled, and a larger craniotomy was made.

### Image analysis

Movement artifacts were corrected for in two steps^61^: a cross correlation-based image alignment (Turboreg)^68^ followed by a line-by-line correction using an algorithm based on a hidden-Markov model^69^. The regions of interest (ROIs) containing the neurons or axonal boutons were then drawn manually (3787 ROIs for MO_s_ layer 2/3, 2321 for V_RL/A/AL_ layer 2/3, 531 for V1 layer 2/3, 2316 for the MO_s_ layer 5, 1199 for V_RL/A/AL_ layer 2/3, 1577 for axonal boutons from the MO_s_, and 760 for axonal boutons from V_RL/A/AL_). The pixel values within each ROI were summed to estimate the fluorescence of the individual neurons/axonal boutons. ∆*F*/*F* signal was calculated as (*F*−*F*_baseline_)/*F*_baseline_, where *F*_baseline_ is the baseline fluorescence signal (30th percentile) within each trial.

Boutons from the same axons were excluded based on correlation analysis among pairs^10,18,36,70^. The correlation coefficients of ∆*F*/*F* during the intertrial interval were calculated for boutons in each plane, and pairs showing a higher correlation (>0.65) were considered to be from the same axon. The high correlation pairs were grouped into clusters^10,18,36,70^, and the ROI with the largest ∆*F*/*F* signal in each cluster was assigned to represent the cluster.

For each neuron or axonal bouton, visual and motor activity were evaluated. Visual activity was quantified as an increase in ∆*F*/*F*, comparing the average ∆*F*/*F* between the control range (−150 ± 150 ms from the onset of the visual stimulus) and the signal range (200 ± 100 ms). Similarly, motor activity was quantified as an increase in ∆*F*/*F*, comparing the average ∆*F*/*F* between the control range (−500 ± 150 ms from the onset of the eye movements) and the signal range (−50 ± 100 ms) (rise time^32^ of GCaMP6f was taken into account). Neurons or boutons were considered to contain visual response if the visual activity was significantly larger than baseline activity (Wilcoxon signed-rank test; *p* < 0.05) in either contraversive or ipsiversive movement conditions. For each neuron, preference for contraversive or ipsiversive conditions was evaluated (Mann–Whitney test; *p* < 0.05). The motor activity was similarly evaluated. To temporally separate visual and motor activity, we rewarded mice for performing with longer reaction times during the training (Supplementary Fig. 8) and analyzed trials with reaction times >1.5 s.

To present the neural activity in the population (heatmaps in Fig. 3e, f, and Fig. 5c, f), neurons showing a significant response either to the visual target or the motor onset were included and realigned in descending order. The activity for each neuron was normalized by its maximum ∆*F*/*F* between 0.5 s before the visual target onset and 0.5 s after the movement onset in the two target conditions.

To quantify the preference for visual and motor information (Fig. 5g–l, Fig. 6c–h, k–p), the stronger response between contraversive and ipsiversive conditions was chosen for visual and motor activity in each neuron and axon terminal and then scatter-plotted for visual (*x*-axis) and motor (*y*-axis) activity. The angle for each point was computed in polar representation, indicating 0 degrees for pure visual activity and 90 degrees for pure motor activity. Similarly the preference for motor information and post-movement activity was quantified (Supplementary Fig. 15). Post-movement activity was evaluated by comparing the average ∆*F*/*F* between the control range (−500 ± 150 ms from the onset of eye movements) and the signal range (400 ± 100 ms).

### Measurement and analysis of whisker movements

The whiskers of three mice were filmed with a CMOS camera (FL3-U3-13Y3M-C, Point Grey) controlled by the custom-made software (written in Visual C++ with the FlyCapture Software Development Kit). Images of the whiskers were saved at 66.6 or 100 Hz with precise frame timing at a pixel resolution of 15.4 × 15.4 μm^2^.

The movement of the whisker was measured by comparing two neighboring frames using a custom-written program in MATLAB. In the series of the images, a small ROI elongated in the anterior–posterior direction containing multiple whiskers was selected. The cross-correlation between neighboring frames was computed for the ROI, and the peak position of the correlation was considered the amount of movement of the whisker position between the two frames (Supplementary Fig. 3).

The relationship between whisking and task structure was evaluated as follows. First, frames with whisker movement >200 μm were identified. If frames were separated by <200 ms, all time points in between were considered the whisking period. The probability of whisking across trials was computed by aligning to the onset of the visual target or eye movement. The probability was compared between the period of −150 ± 150 ms (baseline) and 200 ± 100 ms following the visual target onset and between −500 ± 150 and −50 ± 100 ms before the onset of eye movements.

### Anatomical tracing

In the first experiment (*n* = 3 mice, Supplementary Fig. 6), the subcortical projection patterns of neurons in the MO_s_ were visualized by labeling their axon terminals. A small hole (~250 µm) was made in the skull above the MO_s_ (0.7 mm lateral and 0.7 rostral to the bregma), through which ~50 nl of AAV2/1-EF1α-tdTomato was injected at 300 and 800 µm depths. A few weeks later, the mice were deeply anesthetized (0.3 mg/g ketamine and 0.024 mg/g xylazine, intraperitoneally) and intracardinally perfused with 4% PFA in 0.1 M PBS. The brains were removed, post-fixed in 4% PFA at 4 °C overnight, and rinsed with 0.1 M PBS. The brain was subsequently frozen to be sectioned coronally with a cryotome (40 µm in thickness). Sections were incubated in 0.1 M PBS containing 4% sucrose, 0.05% 3,3’-diaminobenzidine, and 0.05% cytochrome oxidase at 35 °C for 6–8 h. Then the sections were placed on slides in mounting solution (VECTASHIELD HardSet, H-1500, Vector Laboratories). Nuclei borders were drawn based on the signal of cytochrome oxidase reactivity.

In the second experiment (*n* = 3 mice, Fig. 4a–d), the locations of axonal projection patterns from the MO_s_ were identified with the help of callosal axon terminals originating from the contralateral visual area^39–41^. A small hole (~250 µm) was made in the skull above the right MO_s_, through which ~50 nl of AAV2/1-EF1α-tdTomato was injected at 300 and 800 mm depths. Then a 5 mm square of the left cortex was exposed extending between the midline, lambda, and bregma, and 25–30 injections^39^ of ~40 nl of AAV2/1-CAG-GFP were performed at a 300 µm depth. A few weeks later, the mice were perfused and the entire brains were imaged with a fluorescence microscope (Olympus MVX-10 Macroview, 1× MVX Plan Apochromat lens). The filter cube for GFP contained an excitation bandpass filter of 470/40, a dichroic filter of 495, and an emission bandpass filter of 525/50. The filter cube for tdTomato contained an excitation bandpass filter of 545/25, a dichroic filter of 565, and an emission bandpass filter of 605/70. In some cases, the brain regions around the MO_s_ and visual cortex were also imaged using a two-photon microscope with a low-magnification object lens (CFI Plan Fluor 4×, Nikon).

In the third experiment (*n* = 3 mice, Fig. 4e–o), the reciprocal connectivity between the MO_s_ and the visual cortex was examined by sequential virus injections. AAV2/1-EF1α-tdTomato (~50 nl) was injected into the right MO_s_ (at 300 and 800 µm depths) through a small hole (~250 µm) in the skull. After a few weeks, the red signals from the axon terminals could be identified in the visual cortex using a surgical microscope modified with a custom-build light source and filters. Under the guidance of the fluorescence signal from tdTomato, AAV2/1-CAG-eGFP (40 nl) was injected at the axon terminals from the MO_s_ in the visual cortex (depth, 250 µm). A few weeks later, the mice were perfused and the brain was imaged as in the second experiment. The modified surgical microscope had a 530 nm LED (M530L3, Thorlabs) as the excitation light source, with a short pass filter in front of it (575 nm, 84–709, Edmond Optics) and a barrier filter (610 nm, FGL610S, Thorlabs) in front of the objective lens.

### Quantification of anatomical projections

The strength of the anatomical connections between the frontal and parieto-occipital cortex was evaluated using an available database from the Allen Institute for Brain Science^7^ (Supplementary Fig. 12). To quantify projections from the frontal to parieto-occipital cortex, we first set a search filter (source structure: MO_s_ or ACA_d_) to collect available datasets and then downloaded these as a comma-separated values file. We selected the experiments for which the tracer injection site was (1) at 0.5–1.0 mm lateral and 0.5–1.0 mm rostral from the bregma (MO_s_, experiment numbers: 141603190, 168162771, 287995889, 266503540, 272822110, 277957202, and 297892130); (2) at 0–0.5 mm lateral and rostral (ACA_d_, experiment numbers: 125833030, 478491090, 496576666, 478376911, 158018630, 287599685, 526783054, and 177783204); or (3) at 1.0–1.5 mm lateral and rostral (MO_s_, experiment numbers: 181598954, 266176167, 141602484, 288169842, 183617432, 167569313, 168002073, 297669605, 267750528, 156492394, and 267659565, presumed the whisker motor area^71^). Then, we checked axonal density values at ‘VISa’, ‘VISrl’, ‘VISal’, ‘VISp’, or ‘SSbfd’ (corresponding to areas A, RL, AL, V1, and S1 barrel cortex, respectively) for each experiment, normalized them with the largest density value in each experiment, and then compared them. Experiments in (2) were excluded if the injection contained >30% of MO_s_ to reduce contamination. Experiments with interneuron Cre, layer 4 Cre, or layer 6 Cre lines, and those with a labeled sum value <1 were also excluded because long-range projections were rare.

To evaluate projections from the parieto-occipital to the frontal cortex, we similarly set the source structure (VISa, VISrl, VISal, VISp, and SSbfd) to collect available datasets. The datasets were injection (4) at VISa, VISrl, or VISal (*n* = 19 experiments, experiment numbers: 524874308, 518606617, 540145406, 521600943, 303616127, 522635991, 272929308, 518741580, 517325325, 518605900, 120437703, 524666904, 528509838, 297231636, 267761438, 294533406, 510834706, 520996382, 510848595); (5) at VISp (*n* = 12 experiments, experiment numbers: 309113907, 307320960, 307593747, 530574594, 479755622, 307557934, 309003780, 535692871, 531441947, 482578964, 501117182, and 266250195, contamination outside VISp < 2%); and (6) at barrel cortex (*n* = 9 experiments, experiment numbers: 112951804, 100142655, 127866392, 112882565, 297654263, 159602992, 272735030, 100141473, and 175819113, contamination outside SSp-bfd < 50%). Animals with a labeled sum value <1 (1.5 for VISp) were excluded. We then measured axonal projection density for each experiment using coronal sections available at Allen’s website. First, the sections 1.5 mm rostral to the bregma (rostrolateral MO_s_, presumed whisker motor area), 0.75 mm (MO_s_), or 0.25 mm (ACA_d_) were downloaded. Then axonal density was quantified with Image J; ROIs were drawn to cover the MO_s_ or ACA_d_, which matched Allen’s reference atlas shown next to the section, and the mean gray value for each ROI was measured. Finally, each value was normalized with the largest density value in each experiment and then compared with each other.

### Immunohistochemistry

PV was immunostained using standard procedures (Fig. 2b). Coronal slices (thickness, 40 µm) were cut using a cryostat and blocked in carrier solution (5% bovine serum albumin (Sigma-Aldrich); 0.3% Triton X-100 (Sigma-Aldrich) in 0.1 M PBS) for 2 h at room temperature on a shaker. Slices were then incubated with anti-PV primary antibody (1:5000 in carrier solution; Cat. #24428, Hudson, WI, USA) for 18 h at 4 °C on a shaker. After three rinses with 0.1 M PBS for 30 min, sections were incubated with Alexa-Fluor-568-conjugated goat anti-guinea pig secondary antibody (A11075, Invitrogen, 1:500 in carrier solution) for 1 h at room temperature on a shaker. After a few additional rinses for 30 min in 0.1 M PBS, slices were mounted on slide glasses for imaging.

### Statistical analysis

All statistical tests were non-parametric and are indicated in the main text or figure legend. Sample sizes were larger than the minimum size that can yield *p* < 0.05 for each test. Data and traces were show as mean ± s.e.m., unless otherwise stated. Power calculations to predetermine sample sizes were not performed, but our sample sizes are similar to those reported in previous publications^67,72^.

### Code availability

The computer codes from this study are available from the corresponding author upon reasonable request.

### Data availability

The data for this study are available from the corresponding author upon reasonable request.

## Electronic supplementary material

Supplementary Information

Description of Additional Supplementary Information

Supplementary Movie 1
