# Supplementary material for: Streamlined sensory motor communication through cortical reciprocal connectivity in a visually guided eye movement task
Source: Nat Commun. 2018 Jan 23;9:338. doi: 10.1038/s41467-017-02501-4 (PMC5780522; doi:10.1038/s41467-017-02501-4)
Supplement: Supplementary file 1 — Supplementary Information [file 41467_2017_2501_MOESM1_ESM.pdf]

Supplementary Information

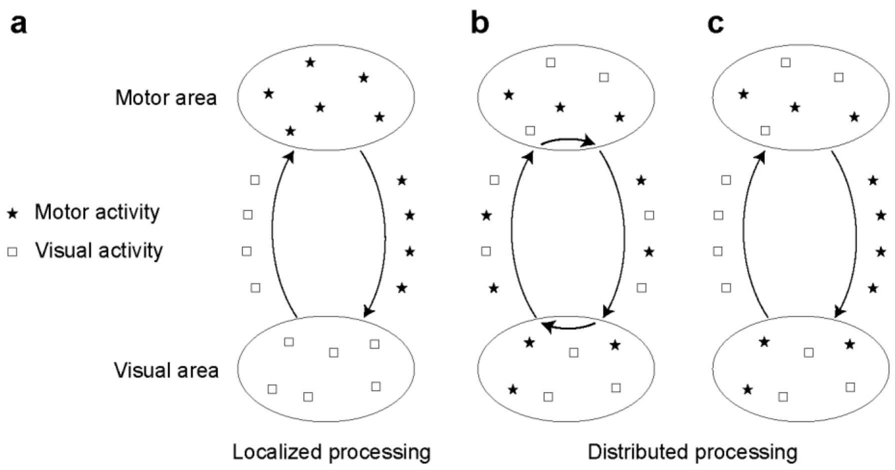

Supplementary Figure 1: Hypothetical network schemes for the communication between sensory and motor areas through reciprocal connectivity. (a) Localized processing. The sensory area exclusively processes sensory information, and the motor area only motor information. (b) Distributed processing with recurrent information flow. The same information may reverberate in the two remote areas. (c) Distributed processing with streamlined information flow. Specific information is selected and transferred depending on the direction of the connections.

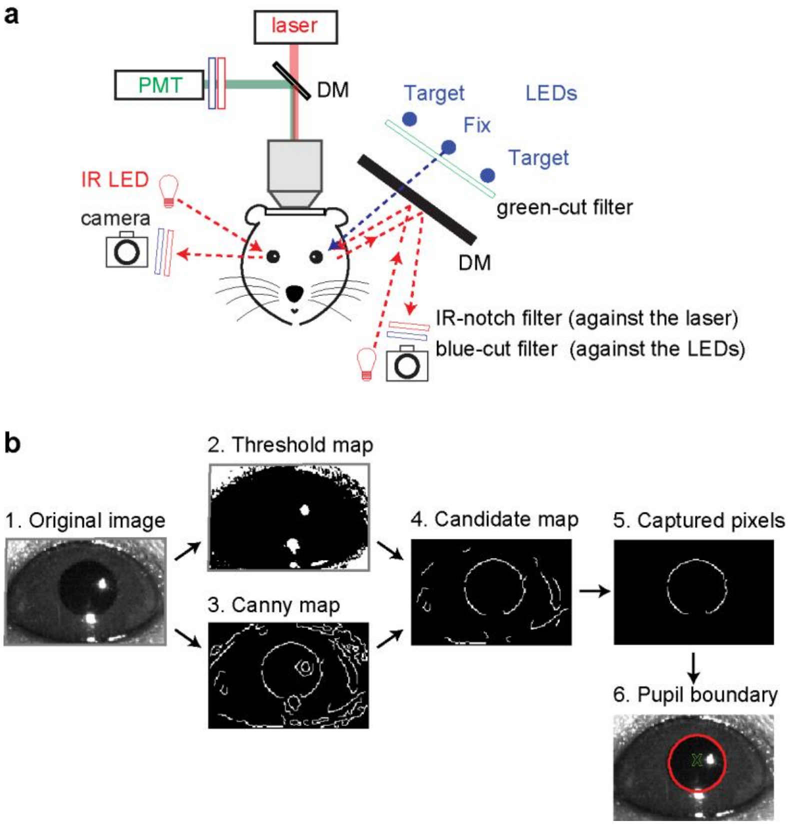

Supplementary Figure 2: Setup and algorithm for detection of eye movements. (a) Optical design for monitoring eye movements. Left eye was illuminated with an IR LED, and filmed with an IR camera. The image of the left eye was reflected in an IR reflecting dichroic mirror. In front of the IR cameras and the photomultiplier tubes (PMTs), a blue-cut filter and an IR-notch filter were placed to block contamination from blue LEDs and the two-photon laser. In front of the blue LEDs, a short-pass filter was placed to prevent contamination at the PMT. In some experiments, the right eye was also filmed. (b) The procedure to detect the boundary of the pupil. Based on the original image (1), two separate processings were applied (2,3) to yield a candidate map (4). Then, the circle that could capture the largest number of white pixels per length (5) was determined (6). See **Methods** for details.

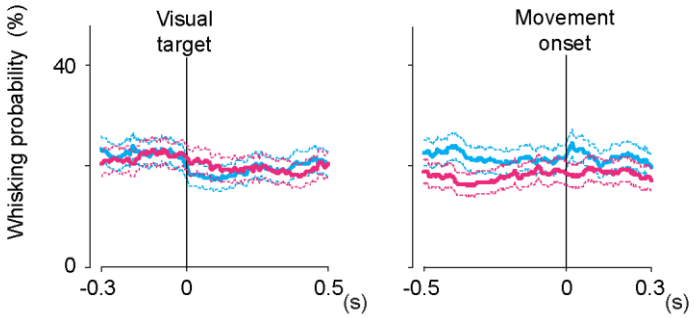

Supplementary Figure 3: Lack of correlation between whisker movements and the task structure of target or movement onset. Averaged probability of the whisking of the left whiskers around the time of the visual target onset and the eye movement onset. There was no significant increase in whisking following the onset of visual targets (nasal, $p = 0.215$, $n = 281$; temporal, $p = 0.071$, $n = 285$ trials, Wilcoxon signed-rank test, three mice) or eye movement onset (nasal: $p = 0.274$; temporal $p = 0.466$). Solid and dashed lines indicate mean ± s.e.m. Magenta, trials with a temporal target; cyan, trials with a nasal target,

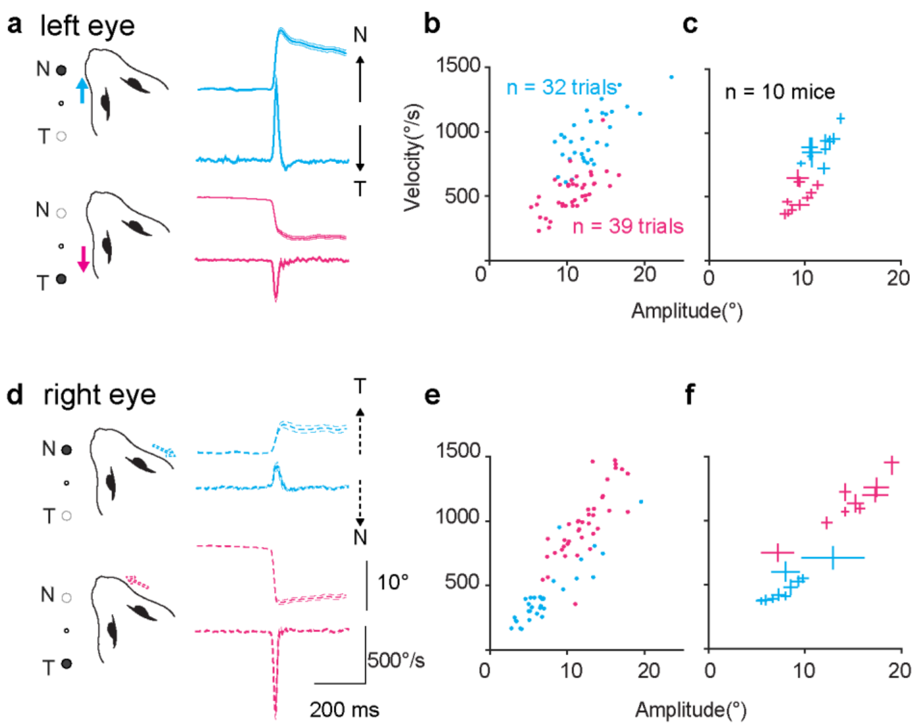

Supplementary Figure 4: Amplitude and velocity of eye movements. (a) Average traces for left eye movements in one behavior session (the same session as Fig. 1e). The position (top) and the velocity (bottom) are shown for nasal target (cyan) and temporal target (magenta) conditions. (b) The relationship between the amplitude and the peak velocity for the left eye in one animal (cyan, nasal target; magenta, temporal). The same session as (a). (c) Similar to b, for ten mice. Each point represents one animal. Error bars indicate s.e.m. (d-f) Same as a-c for the right eye.

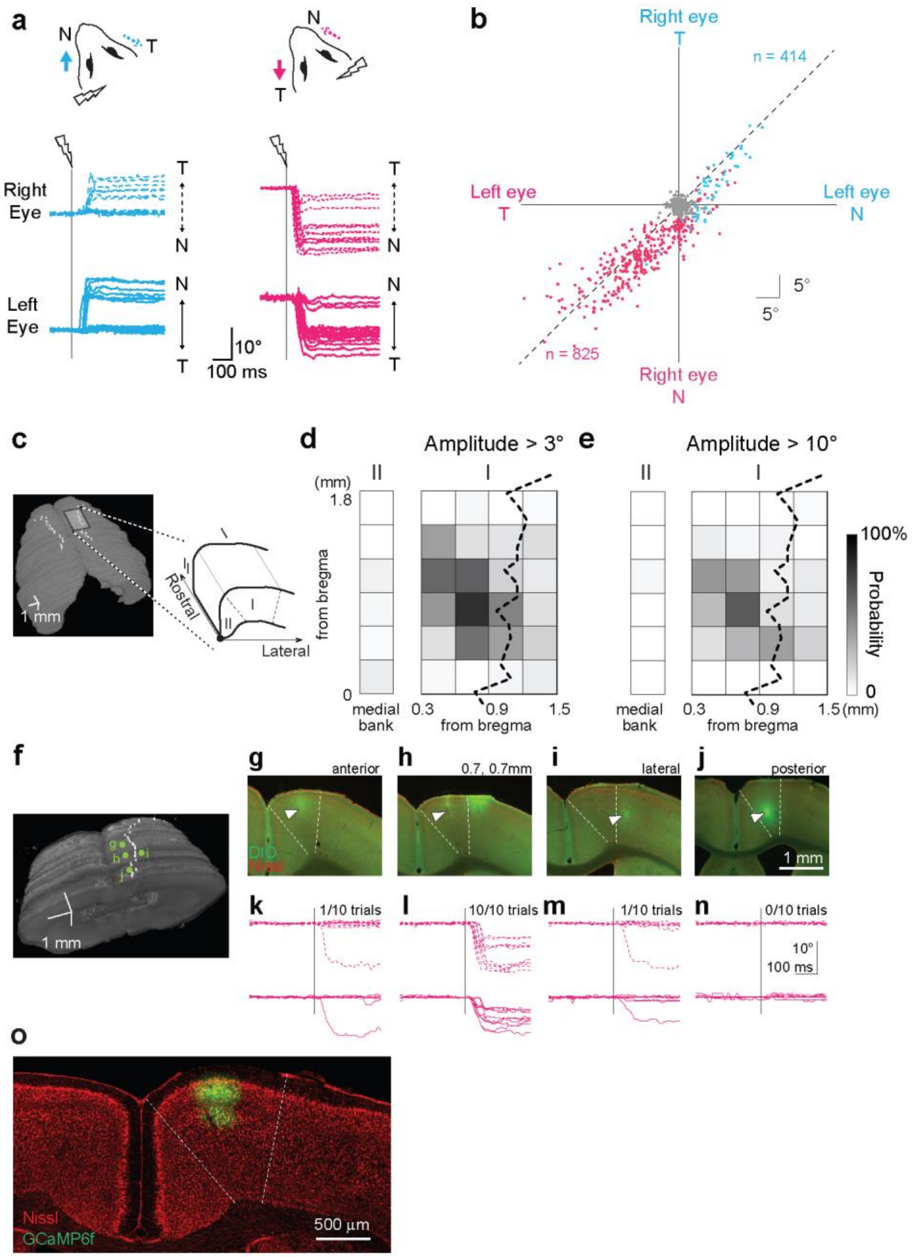

Supplementary Figure 5: Identification of a cortical area responsible for eye movements based on microstimulation. (a,b) Electrical microstimulation of the frontal cortex evoked binocularly coupled eye movements. (a) Electrical microstimulation at one location in the left frontal cortex evoked a binocular eye movement toward the right side. Microstimulation of the right frontal cortex evoked a binocular movement toward the left side. Stimulation parameters: amplitude, 20-50 μA; frequency, 200Hz; pulse width, 300 μs; duration, 250 ms (b) The amplitudes of the eye movements for two eyes are shown when the stimulation was in the left (n = 414 stimulations, 26 sites, cyan) or right (n = 825 stimulations, 62 sites, magenta) hemisphere. Amplitudes below 3 degrees were considered as a failure (gray points). (c-e) Eye movements were induced when the microstimulation site was in a small portion of the secondary motor cortex. (c) Three-dimensional view of the cortex reconstructed from Paxinos and Franklin's atlas[1]. The black rectangle indicates the microstimulated area in d and e. Stimulation electrode was advanced to the infragranular layer (see Methods). The white line indicates the border between the secondary and the primary motor cortex. (d) The probability of eliciting an eye movement at each stimulation site (n = 12 animals). The x-axis indicates distance from the mid-sagittal line for the dorsat part (I), and the y-axis indicates rostrocaudal distance from the bregma for both the dorsal part (I) and the medial bank (II). Stimulation sites in both hemispheres are included. The

border between the secondary and the primary motor cortex, expected from Paxinos and Franklins's atlas[1], is indicated as a black dotted line. (e) The probability of eliciting eye movements larger than 10 degrees. (f-n) One example of a microstimulation. The border of the secondary motor cortex[2] (white lines) was determined based on thinner supragranular and infragranular layers in the corresponding and the neighboring sections. Green dots indicate the coordinates of the four insertions shown in g-j. (g-j) The electrode was inserted at 1.3 mm and 0.7 mm rostrolateral to the bregma (labelled as anterior, g), 0.7 mm and 0.7 mm (as center, h), 0.6 mm and 1.3 mm (as lateral, i), and 0.1 mm and 0.7 mm (as posterior, j). Arrow heads indicate electrode tracks. (k-m) Eye position traces following the stimulation at sites g-j. Success rates in eliciting eye movements are indicated at the top right. The stimulation was ten times per site. (o) Nissl-stained coronal sections at coordinates 0.7mm rostrolateral to the bregma. Injection of GCaMP6f AAV virus were confirmed to be in the secondary motor cortex.

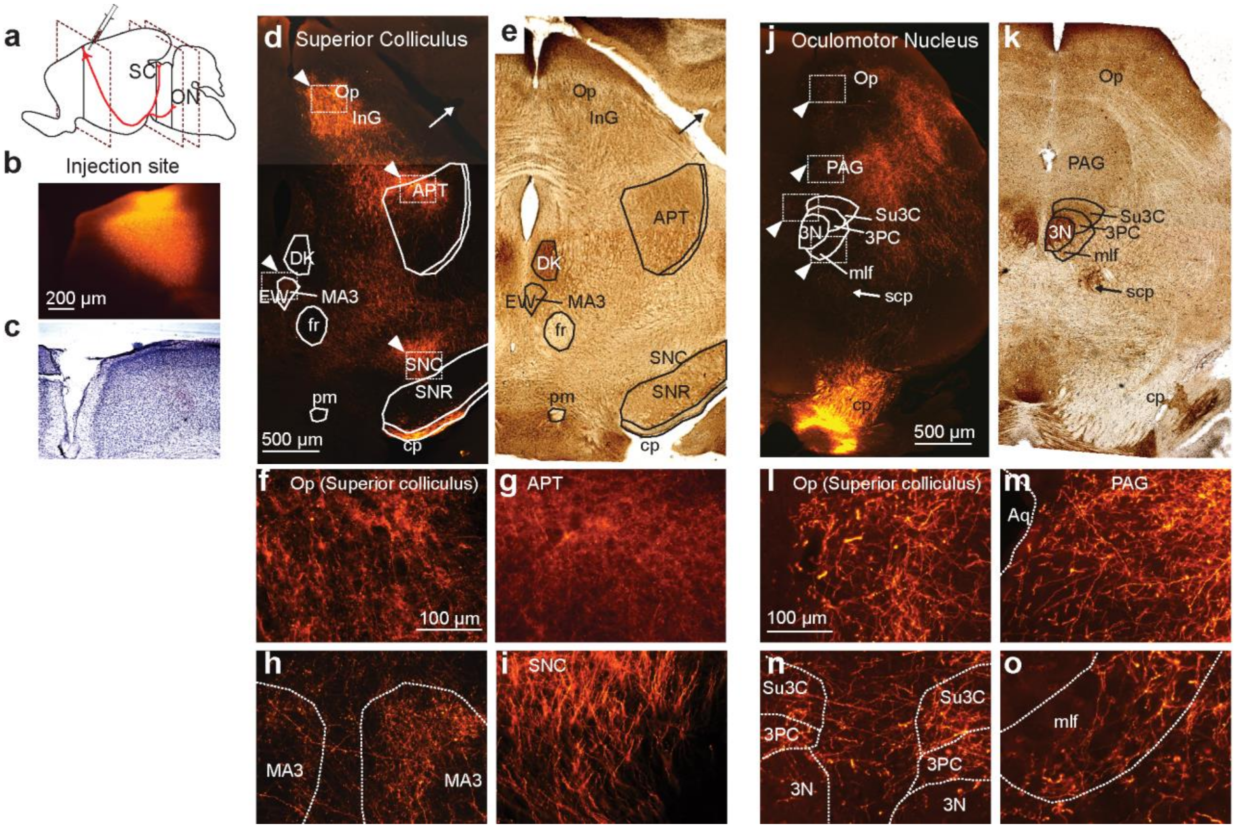

Supplementary Figure 6: MO_s neurons project to nuclei and areas for eye movements at midbrain. (a-c) The experimental design. (b) The injection site of AAV-EF1α-tdTomato. (c) An adjacent section to b with Nissl staining. (d-i) Section at the level of the rostral superior colliculus. (e) Adjacent section to d, processed for cytochrome oxidase reactivity to show the different nuclei and tracts. (f-i) Magnified images shown in d. (j-o) Similar to d-i, but at the level of the oculomotor nucleus. APT: anterior pretectal nucleus, Aq: aqeduct, cp: cerebral peduncle basal part, EW: Edinger-Westphal nucleus, DK: nucleus of Darkschewitsch, fr: fasciculus retroflexus, InG: intermediate gray layer of the superior colliculus, MA3: medial accessory oculomotor nuclei, mlf: medial longitudinal fasiculus, Op: optic nerve layer of the superior colliculus, PAG: periacqueductal gray, pm: principal mammillary tract, scp: superior cerebellar peduncle, SNC: substantia nigra pars compacta, SNR: substantia nigra pars reticulata, Su3C: supraoculomotor caps, 3N: oculomotor nucleus, 3PC: oculomotor nucleus parvicellular part.

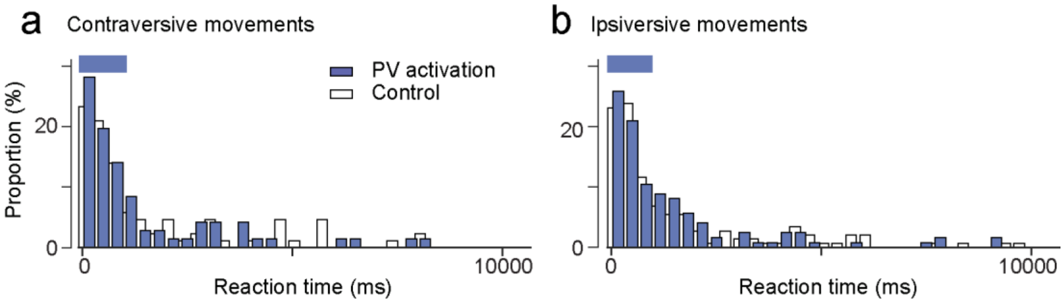

Supplementary Figure 7: Optogenetic suppression of the primary motor cortex during the visually guided eye movement task. (a,b) Distribution of the reaction times for the eye movements. PV activation had only minor effects on both contraversive eye movements (a, PV activation, n= 71 trial; control, n= 86 trials; p = 0.95) and ipsiversive movements (b, PV activation, n = 124 trials; control, n = 147 trials; p = 0.90, Person’s chi-square test).

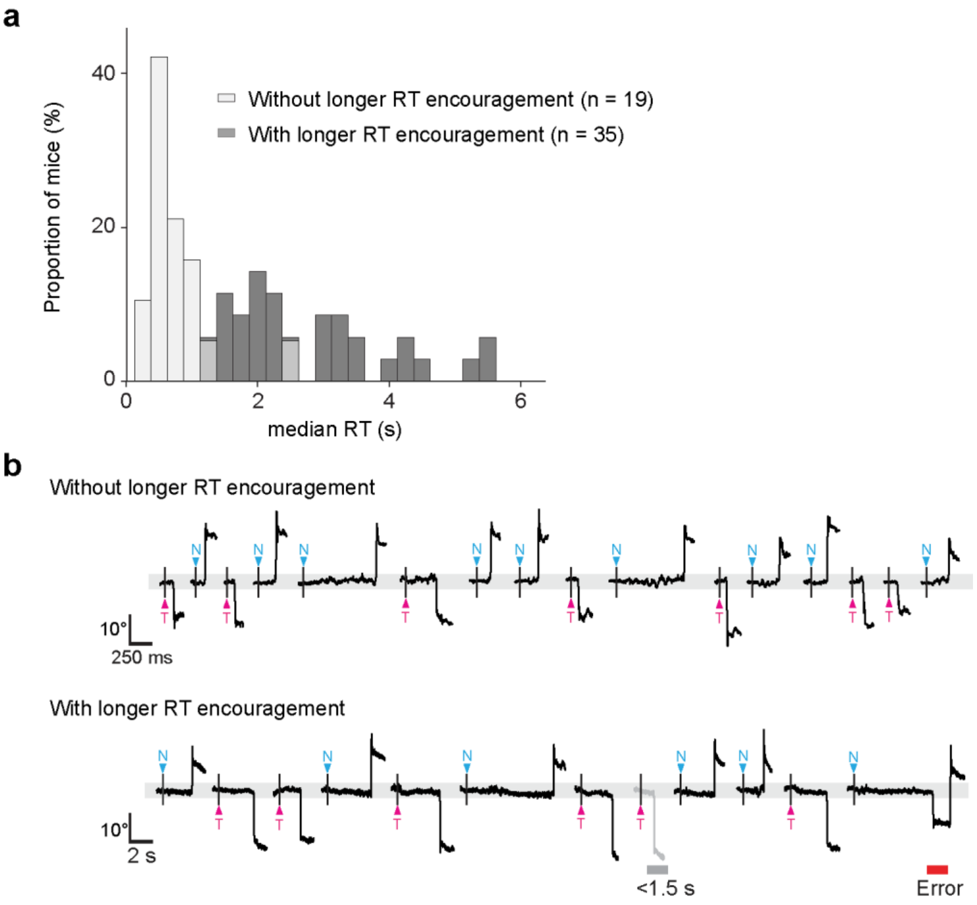

Supplementary Figure 8: For imaging experiments, mice were encouraged to make eye movements with a longer reaction time. (a) Distribution of median reaction time without and with encouraging longer reaction times (19 and 35 mice). Success: 85.9 ± 2.2%, no eye movement: 8.9 ± 1.4%, and incorrect eye movements: 5.3 ± 1.2% without the encouragement (n = 19 mice), and 80.4 ± 1.6%, 6.1 ± 0.8%, and 13.5 ± 1.3% with the encouragement (n = 35). (b) Example traces of eye position for two mice without or with longer reaction time encouragement. Arrows with N indicate the stimulus onset for the nasal target; T indicates temporal. Note the time scale is longer in the bottom traces.

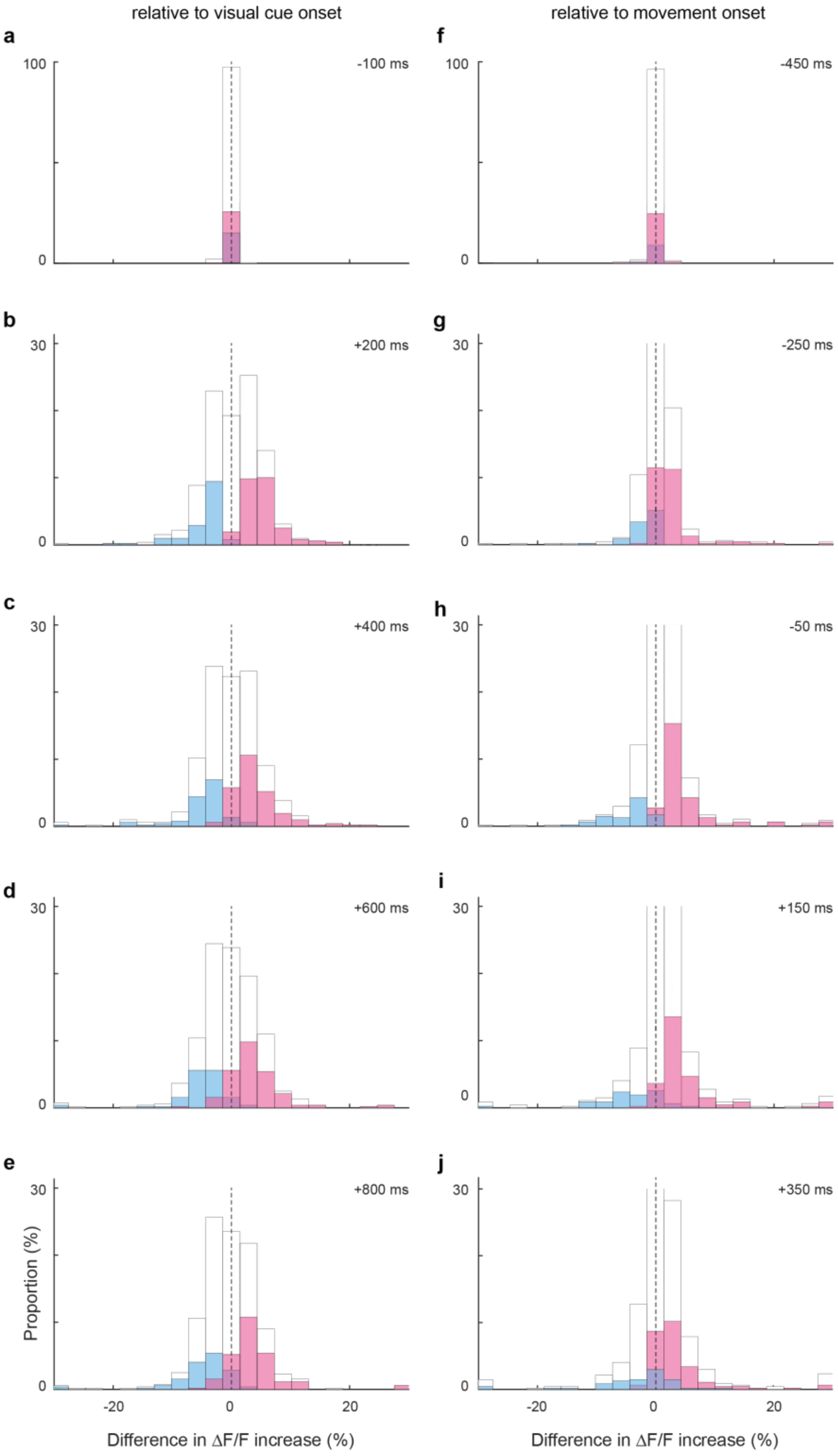

Supplementary Figure 9: Time-course of the laterality in visual and motor activity in the MOs. (a-e) Histograms of the difference in visual activity between ipsiversive and contraversive conditions. Magenta, neurons that exhibited significantly larger visual activity in contraversive conditions (n = 135, statistics based on 200 ± 100 ms following the visual target onset, see Methods); cyan, neurons in ipsiversive conditions (n = 80). (f-j) Same as (a-e) but for motor activity. Magenta, n = 122; cyan, n = 45, based on -50 ± 100 ms from the movement onset.

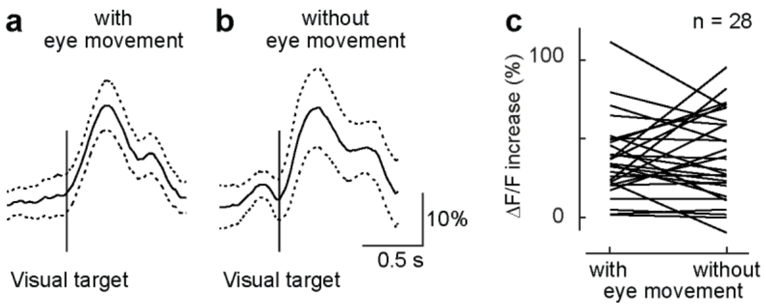

Supplementary Figure 10: Response to a visual target without subsequent eye movements. (a, b) Response of one example cell to the temporal visual target. Eye movements followed in 17 trials (a), but not in six trials (b). (c) Comparison of visual responses between trials with and without subsequent eye movements (n = 28 neurons from two imaging sessions). No significant difference was found (p = 0.50, Wilcoxon signed-rank test). Note that most errors were incorrect eye movements, so only these two sessions had more than five errors of no eye movement for a single target.

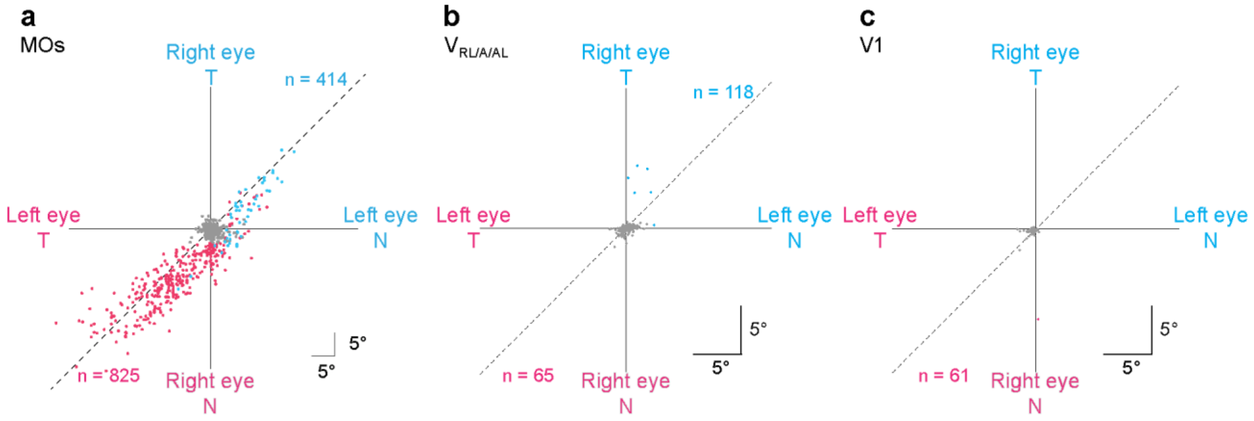

Supplementary Figure 11: Microstimulation of the MO_s, $V_{RL/A/AL}$, and V1. (a) Stimulation of the MO_S, redrawn from Supplementary Fig. 5b. The amplitude of the eye movement for the two eyes is shown when the stimulation was in the left (n = 414 stimulations, 26 sites, cyan) or right (n = 825 stimulations, 62 sites, magenta) hemisphere. (b) The same is shown for the $V_{RL/A/AL}$, which was identified based on the projections from the MO_S The stimulation was in the left (n = 118 stimulations, 6 sites) or right (n = 65 stimulations, 5 sites) hemisphere. (c) The same is shown for the V1. The stimulation was in the right hemisphere (n = 61 stimulations, 6 sites).

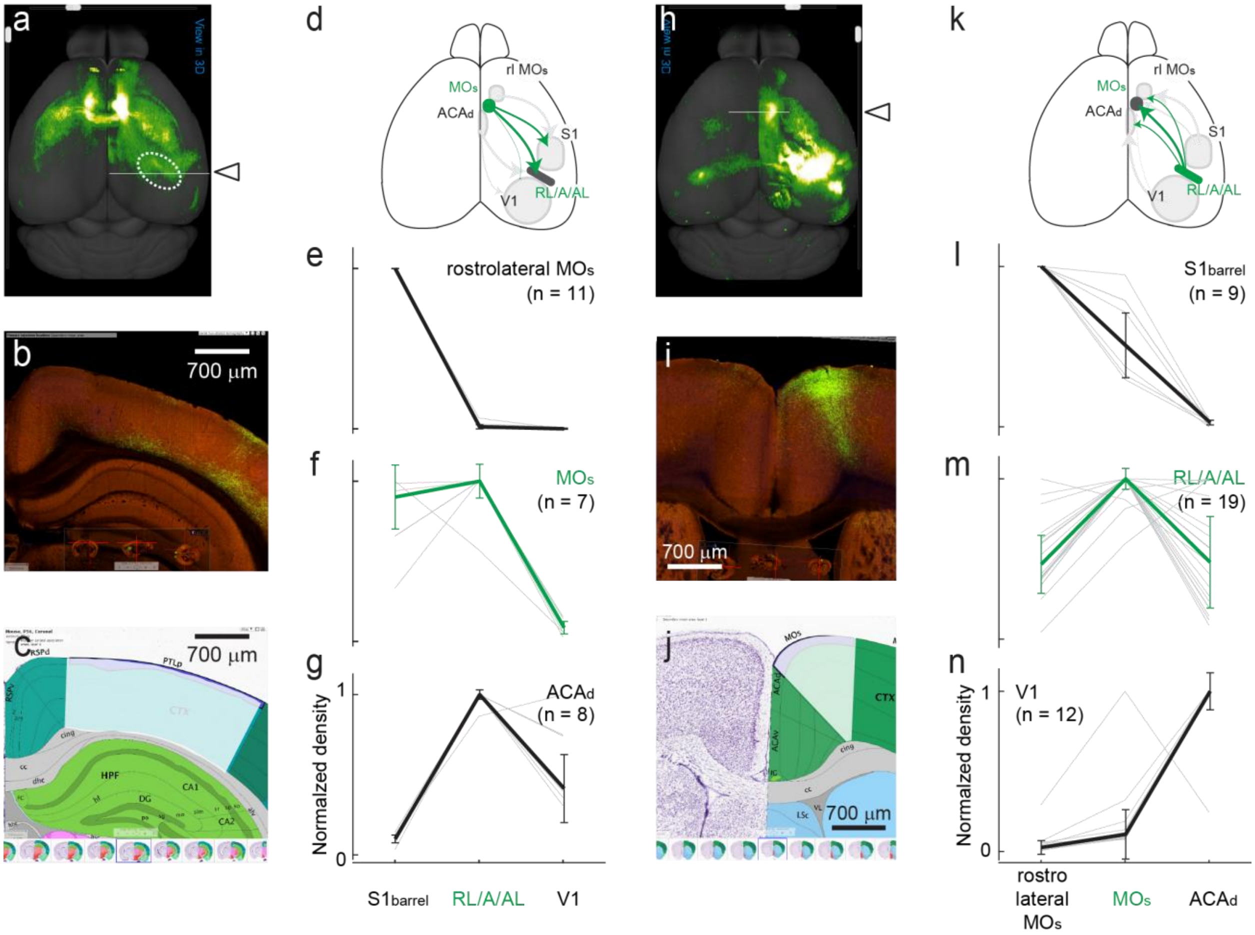

Supplementary Figure 12: Reciprocal connectivity between the frontal and parieto-occipital cortices. (a-g) Projections from the frontal to parieto-occipital cortex. (a-c) One example dataset from the Allen Institute for Brain Science where GFP virus was injected into the secondary motor cortex (experiment #266503540). Note a prominent band in the parieto-occipital region (dotted ellipse in a). (b) A coronal section at the parieto-occipital region from the dataset shown in a (rostro-caudal level shown as the arrowhead). (c) Best-matched atlas section to the section b selected by Allen's program. The 'posterior parietal association areas (PTLp)', consisting of areas RL and A, was marked in white. (d-g) Population analysis based on datasets from the Allen Institute for Brain Science (see **Methods**). As the injection site, the rostrolateral MO_s (e, overlapping with presumed whisker motor area[5]), MO_s (f), and ACA_d (g) were selected, and axonal density in S1 barrel cortex, areas RL/A/AL, and V1 were evaluated. As summarized in d, the MO_s sends strong projections to areas RL/A/AL and the S1 barrel cortex, whereas rostrolateral MO_s sends to the S1 barrel cortex and ACA_d to areas RL/A/AL. Individual experiments were shown in thin gray lines, and their median ± median absolute deviation was shown in a thick line. Friedman test ($p<0.05$) followed by Wilcoxon signed-rank test (b, n = 11 experiments, $p_{\text{S1barrel vs RL/A/AL}} < 10^{-3}$, $p_{\text{RL/A/AL vs V1}} < 0.01$, $p_{\text{S1barrel vs V1}} < 10^{-3}$; c, n = 7, $p_{\text{S1barrel vs RL/A/AL}} = 0.219$, $p_{\text{RL/A/AL vs V1}} = 0.0156$, $p_{\text{S1barrel vs V1}} = 0.0156$; d, n = 8, $p_{\text{S1barrel vs RL/A/AL}} < 0.01$, $p_{\text{RL/A/AL vs V1}} = 0.0156$, $p_{\text{S1barrel vs V1}} < 0.01$). Statistical significance for Wilcoxon signed-rank test is 0.0167 (Bonferroni correction). (h-n) Projections from the parieto-occipital to the frontal cortex. (h-j) Similar to a-c, for a dataset where virus was injected into the area RL (experiment #521600943). Images h and i were horizontally reflected to match the laterality of the virus injection. (i) A coronal section (rostro-caudal level shown as the arrowhead in h). (j) Automatically selected atlas section. The secondary motor cortex was marked in white. (k-n) Population analysis. As the injection site, the S1 barrel cortex (l), areas RL/A/AL (m), and V1 (n) were selected, and axonal density in the MO_s,

rostrolateral MO_s, and ACA_d were evaluated. As summarized in k, areas RL/A/AL send strong projections to the MO_s, whereas S1 barrel cortex sends to the rostrolateral MO_s and the V1 to the ACA_d. Friedman test ($p < 0.05$) followed by Wilcoxon signed-rank test (l, n = 9 experiments, $p_{\text{rlMOs vs MOs}} < 0.01$, $p_{\text{MOs vs ACAd}} < 0.01$, $p_{\text{rlMOs vs ACAd}} < 0.01$; m, n = 19, $p = {}_{\text{rlMOs vs MOs}} < 10^{-3}$, $p_{\text{MOs vs ACAd}} < 0.01$, $p_{\text{rlMOs vs ACAd}} = 0.841$; n, n = 12, $p_{\text{rlMOs vs MOs}} < 10^{-3}$, $p_{\text{MOs vs ACAd}} < 0.01$, $p_{\text{rlMOs vs ACAd}} < 10^{-3}$).

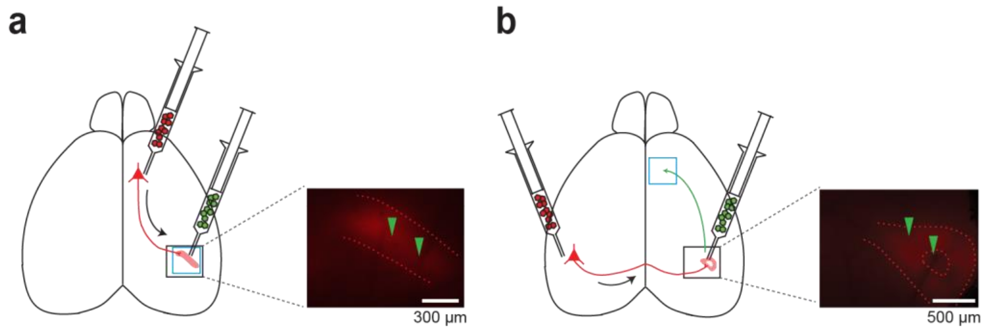

Supplementary Figure 13: Identification of the $V_{RL/A/AL}$ for GCaMP6f virus injection through skull. For the virus injection, the location of the $V_{RL/A/AL}$ was identified with axon terminals from the ipsilateral MO_s (a) or the contralateral visual areas (b). These terminals were labeled with AAV-tdTomato or dextran Alexa 594 (see Methods), and could be identified through thinned skull (images in a, b). Then, AAV-GCaMP6f was injected into the terminals for somatic calcium imaging in the $V_{RL/A/AL}$ (green arrows in a) or axonal calcium imaging in the MO_s (b). Imaging sites are indicated as cyan squares in the diagrams.

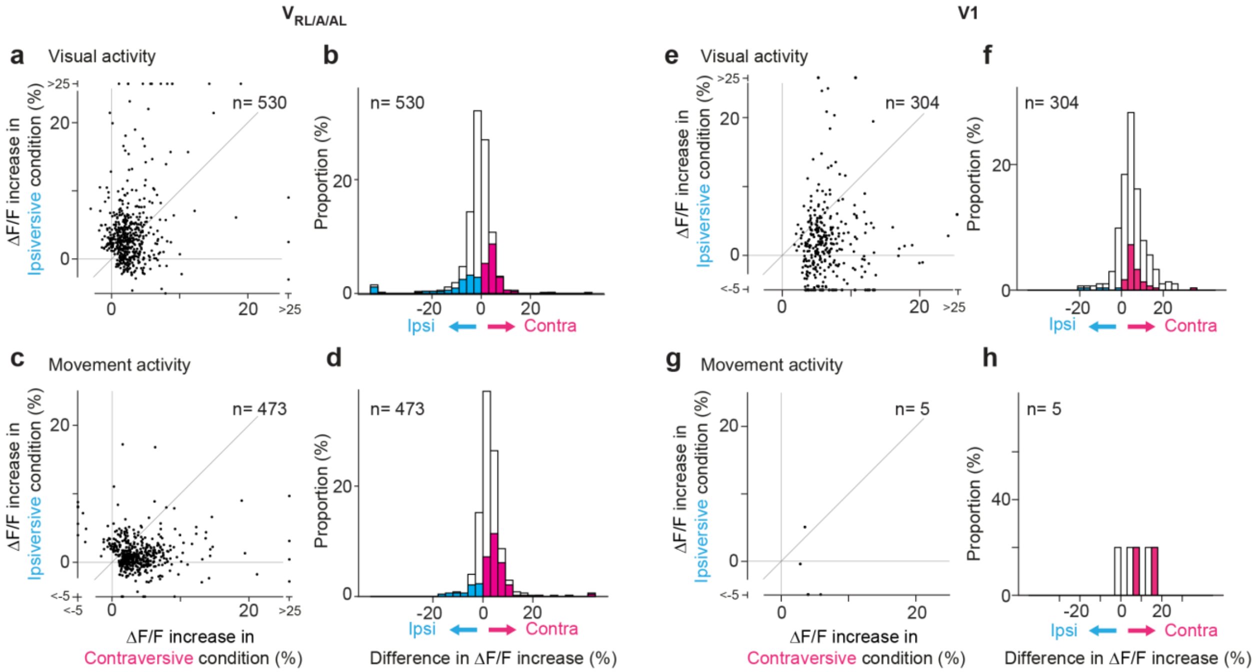

Supplementary Figure 14: Laterality of neural coding in the $V_{RL/A/AL}$ and V1. (a-d) Coding in the $V_{RL/A/AL}$. (a) Comparison of visual responses between trials for the contraversive or ipsiversive movements (n = 530). (b) A histogram for the difference in the visual responses between the two conditions (n = 530). Magenta histogram, 95 neurons with stronger responses in the contraversive condition ($p < 0.05$, Mann-Whitney test); cyan, 67 neurons with stronger responses in the ipsiversive condition ($p < 0.05$). (c,d) Similar to a,b, but for movement activity (n = 473). Magenta, 133 neurons; cyan, 24 neurons. (e-h) same as a-d for the V1 (n = 304 neurons, visual activity: magenta, 45 neurons, cyan, five neurons, motor activity: magenta, two neurons, cyan: 0 neurons).

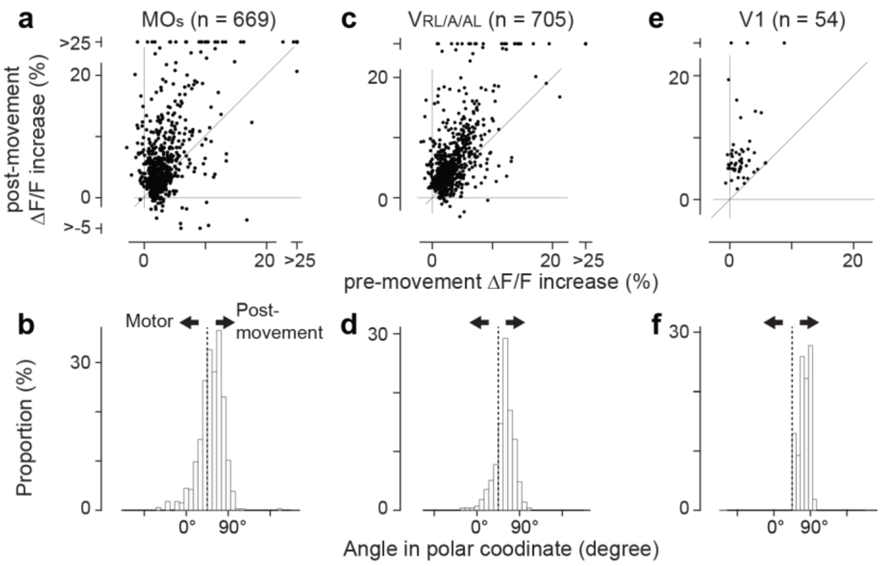

Supplementary Figure 15: Comparison between motor and post-movement activity in MO_s, $V_{RL/A/AL}$ and V1. (a,c,e) Motor and post-movement activity was plotted for each neuron in the MO_s (a, n = 669), $V_{RL/A/AL}$ (c, n = 705), and V1 (e, n = 54). Neurons showing significant motor or post-movement activity are included. (b,d,f) The angle in polar coordinates was computed for each neuron, and plotted as a histogram. Degrees of 0 or 90 indicate pure motor or post-movement activity.

Supplementary Movie 1: Binocularly coupled eye movements during the task. The two eyes were monitored and filmed with IR cameras. The image of the left eye is shown on the left, that of the right on the right for a representative animal (six trials). The movie clips were slowed down to 0.25x for better visualization. The white square indicates the onset timing and the position of the visual targets. Note that the movement of the two eyes were in the same direction and highly synchronized. N: nasal side, T: temporal side.

1 Paxinos, G. & Franklin, K. *The Mouse Brain in Stereotaxic Coordinates. second edition*. (Academic Press, 2001).

2 Brecht, M. *et al.* Organization of rat vibrissa motor cortex and adjacent areas according to cytoarchitectonics, microstimulation, and intracellular stimulation of identified cells. *J. Comp. Neurol.* **479**, 360-373, doi:10.1002/cne.20306 (2004).

3 Tohmi, M., Meguro, R., Tsukano, H., Hishida, R. & Shibuki, K. The extrageniculate visual pathway generates distinct response properties in the higher visual areas of mice. *Curr. Biol.* **24**, 587-597, doi:10.1016/j.cub.2014.01.061 (2014).

4 Takahashi, T. The organization of the lateral thalamus of the hooded rat. *J. Comp. Neurol.* **231**, 281-309, doi:10.1002/cne.902310302 (1985).

5 Sreenivasan, V. *et al.* Movement Initiation Signals in Mouse Whisker Motor Cortex. *Neuron* **92**, 1368-1382, doi:10.1016/j.neuron.2016.12.001 (2016).
