# Supplementary material for: Streamlined sensory motor communication through cortical reciprocal connectivity in a visually guided eye movement task
Source: Nat Commun. 2018 Jan 23;9:338. doi: 10.1038/s41467-017-02501-4 (PMC5780522; doi:10.1038/s41467-017-02501-4)
Supplement: Supplementary file 2 — Description of Additional Supplementary Information [file 41467_2017_2501_MOESM2_ESM.pdf]

Description of Additional Supplementary Files

File Name: Supplementary Movie 1
Description: Binocularly coupled eye movements during the task. The two eyes were monitored and filmed with IR cameras. The image of the left eye is shown on the left, that of the right on the right for a representative animal (six trials). The movie clips were slowed down to 0.25x for better visualization. The white square indicates the onset timing and the position of the visual targets. Note that the movement of the two eyes were in the same direction and highly synchronized. N: nasal side, T: temporal side.
